# Effects of voluntary wheel running on appetite-regulating peptides and neuroinflammation in the hypothalamus of ovariectomized middle-aged mice

**DOI:** 10.3389/fnmol.2025.1698306

**Published:** 2025-12-08

**Authors:** Mateusz Grabowski, Konstancja Grabowska, Magdalena Kostka, Natalia Pondel, Halina Jędrzejowska-Szypułka, Andrzej Małecki, Jarosław J. Barski, Marta Nowacka-Chmielewska

**Affiliations:** 1 Laboratory of Molecular Biology, Institute of Physiotherapy and Health Sciences, Academy of Physical Education, Katowice, Poland; 2 Department of Physiology, Faculty of Medical Sciences in Katowice, Medical University of Silesia, Katowice, Poland

**Keywords:** appetite peptides, hypothalamus, neuroinflammation, inflammasome NLRP3, voluntary wheel running, physical activity, ovariectomy, menopause

## Abstract

The postmenopausal period is associated with an increased tendency to gain weight. This may be due to disturbances in appetite regulation, mainly in the hypothalamus and nutritional behaviors, as well as persistent neuroinflammation resulting from estrogen deficiency. Research indicates that physical activity may counteract estrogen deficiency by improving hypothalamic regulation of appetite and inflammation, thereby promoting better energy balance and decreasing the risk of weight gain after menopause. We investigated whether voluntary wheel running (VWR) impacts factors related to appetite, energy homeostasis, and neuroinflammatory changes induced by ovariectomy (OVX). Thus, 13-month-old female mice underwent OVX, creating a comprehensive model of reproductive aging in females. Ovariectomized (OVX-VWR) and sham-operated (SHAM-VWR) mice were subjected to 6 weeks of VWR. The control sedentary groups (SHAM-SED, OVX-SED) were housed with immobilized wheels. The body mass, food and water intake, and daily running activity were recorded. Hypothalamic and serum samples were collected to examine the expression levels of genes, proteins, and hormones related to appetite regulation and neuroinflammation processes. OVX mice gained weight most excessively and showed reduced running activity. OVX downregulated the ERα/ERβ ratio, and VWR increased ERβ expression. VWR increased Lepr and Cckar expression in the sham-operated group. VWR has an impact on hypothalamic neuroinflammation regardless of ovarian status, through changes in expression of NLRP3, pro-IL-18, TLR4, pro-caspase 1, *Il-1b*, and *Il-18*. OVX in middle-aged mice altered body weight and energy metabolism, but did not affect food intake. VWR modulated hypothalamic appetite-regulating factors—changes not seen in OVX females—and elicited a comparable neuroinflammatory response in the hypothalamus of both SHAM- and OVX-operated mice.

## 1 Introduction

Estrogens play a crucial role not only in regulating the development and functions of the reproductive system but also in influencing other body systems. Clinical and experimental studies indicate that estrogens may affect several pathways related to energy homeostasis, including thermoregulation, energy metabolism, appetite regulation, energy expenditure, insulin and leptin sensitivity, and body fat distribution (Yang et al., 2020; Ainslie et al., 2001; Huang et al., 2017; Tang et al., 2022). During the perimenopausal period, changes in adipose tissue metabolism occur due to the marked decrease in circulating estrogen levels. This decline makes women more susceptible to weight gain and may increase the risk of visceral obesity, which is associated with various cardiometabolic complications and hormone-related malignancies (Farahmand et al., 2022; Ahmed et al., 2022; Zivkovic et al., 2011; Slattery et al., 2015). Estrogens have been found to regulate appetite by activating anorexigenic neurons, leading to hunger suppression, or by inhibiting orexigenic neurons, thereby promoting appetite (Zhu et al., 2023). Therefore, estrogen deficiency can disrupt appetite regulation (Soni et al., 2011), potentially by altering neuronal activity, which may result in increased hunger and food consumption (Stincic et al., 2018; Olofsson et al., 2009). Furthermore, obesity that may arise from increased caloric intake can overlap with the systemic and central hormonal changes associated with estrogen deficiency during postmenopause (Vigil et al., 2022).

Postmenopause may be associated with chronic low-grade inflammation in both peripheral organs and the central nervous system (CNS) (Sárvári et al., 2012; Sadashiv et al., 2015; Ter Horst et al., 2020). Disrupted signaling of appetite-regulating factors has been associated with neuroinflammation in the hypothalamus (Cui et al., 2024; Oberto et al., 2022). Furthermore, this hypothalamic neuroinflammation, partly reactive gliosis, has been linked to neurodegeneration and metabolic disorders, including obesity (Sanchez et al., 2024; Goo et al., 2025; Kim et al., 2019; Cocozza et al., 2021; Thaler et al., 2012). Estrogens in the CNS exert neuroprotective effects, in part by modulating neuroinflammatory processes (Yilmaz et al., 2019). Experimental studies suggested that the cessation of ovarian function leads to hypothalamic microglial activation, accompanied by the upregulation of several genes involved in inflammatory responses, which may significantly contribute to the disruption of energy homeostasis (Baek et al., 2024; Yang et al., 2020). As women increasingly spend a larger portion of their lives in postmenopause, the health implications and potential impacts on their quality of life become increasingly important as one of the major public health concerns (Opoku et al., 2023). Therefore, to assess the changes in neuroinflammatory and central appetite-related factors associated with postmenopause, we introduced the established mouse menopause model via ovariectomy in middle-aged mice. Moreover, previous research demonstrated that ovariectomy seems to trigger the onset of overweight conditions that mimic menopause-related obesity (Yang et al., 2020).

Introducing lifestyle changes, such as increased physical activity, may exert various beneficial effects at different levels, from influencing cellular signaling pathways to extending lifespan (Gremeaux et al., 2012). Moreover, physical activity reduces the risk of development, and it is considered to prevent not only age-related disorders (Chodzko-Zajko et al., 2009; Zhao et al., 2024; Galle et al., 2023; Dishman et al., 2021). In middle-aged women, physical activity may prevent cardiovascular problems (Azarbal et al., 2014) and abdominal obesity, favorably influence lipid profile (Hyvärinen et al., 2022; de Araújo et al., 2023), and modulate energy expenditure after weight loss (Hunter et al., 2015). Furthermore, physical exercise promotes weight loss and total body fat loss in normal-weight and overweight/obese individuals (Dupuit et al., 2020; Dupuit et al., 2022). Exercise-induced weight control in obese middle-aged women was positively associated with changes in appetite-regulating hormones, partly due to its impact on neuroinflammation (Ropelle et al., 2021) and improved insulin resistance (Kang et al., 2018; Liu et al., 2024). Physical activity has demonstrated effectiveness in decreasing neuroinflammatory markers, such as tumor necrosis factor alpha (TNF-*α*), and in modulating microglial activation in the hypothalamus, which may restore central appetite regulation and energy balance homeostasis (Sasaki et al., 2024; Gonzalez-Gil and Elizondo-Montemayor, 2020). Voluntary wheel running was demonstrated to enhance insulin sensitivity, modulate the immune and metabolic profile of white adipose tissue, and have positive effects on hypothalamic inflammation in obese mice. Furthermore, these studies indicate favorable alterations in the expression of neuropeptides and hormones associated with appetite regulation (Sasaki et al., 2024; Zidon et al., 2018; Appleyard et al., 2021; Soch et al., 2016; Ruegsegger et al., 2015). Previous studies have shown that voluntary wheel running mitigates metabolic inflammation in adipose tissue after ovariectomy (Zidon et al., 2018) and that hypothalamic neuropeptide signaling, particularly Npy expression, correlates with wheel running behavior and energy homeostasis (Ruegsegger et al., 2016). The present study expands upon these findings by focusing on intermediate-aged mice to explore the neuroinflammatory and behavioral consequences of ovarian hormone deprivation, thus providing translational relevance to the perimenopausal period in women.

Our previous study demonstrated age-dependent neuroinflammatory responses to voluntary wheel running and metformin treatment in the frontal cortex of ovariectomized mice (Grabowska et al., 2025). In the present study, we extend these findings by focusing on the hypothalamus. We hypothesize that bilateral ovariectomy induces appetite disruption related to neuroinflammation changes in the hypothalamus of middle-aged mice. These changes may underlie the tendency toward weight gain and metabolic disturbances characteristic of postmenopause. Therefore, a key issue is to determine whether voluntary wheel running may impact the appetite, metabolic, and neuroinflammatory ovariectomy-induced changes. This study focuses on the modulation of hypothalamic protein and gene expression levels involved in orexigenic and anorexigenic signaling pathways, as well as inflammation, including NLR family pyrin domain containing 3 (NLRP3) inflammasome activation. The findings open up a discussion about the role of physical activity in the crosstalk of the neuroinflammation process and appetite control in the postmenopausal period.

## 2 Materials and methods

### 2.1 Animals

Nulliparous female C57BL/6 N mice were obtained from the Animal House of the Medical University of Silesia in Katowice, Poland. The animals were treated following protocols approved and monitored by the Local Committee for the Care and Use of Laboratory Animals in Katowice, Poland (permission no. 61/2020, 30th November 2020). The study was conducted in accordance with the ARRIVE 2.0 guidelines and the 3Rs principles (Replacement, Reduction, and Refinement) to ensure ethical and transparent reporting of animal research. We used the minimum number of mice necessary to obtain consistent data, and every effort was made to minimize the suffering of the animals. The mice were housed in a climate-controlled environment (22 ± 1 °C, 55 ± 10% humidity) with a 12-h light/dark cycle (lights on at 07:00). The phase of the estrus cycle was assessed during the light phase. Before the experiments, animals were kept in groups of 5–8 per cage in standard polycarbonate cages (42.5 × 26.6 × 15.5 cm; model 1, Tecniplast 1,285 L, Buguggiate, Italy) equipped with an integrated running wheel (diameter 11 cm; ClockLab, ActiMetrics, Wilmette, IL, USA), which also provided environmental enrichment.”

### 2.2 Experimental design

The study included 40 middle-aged female mice (13 months old), randomly divided into two groups. Animals in the first group were subjected to bilateral ovariectomy (OVX, *n* = 20), while the other group underwent a sham ovariectomy without removing ovaries (SHAM, *n* = 20). After operations, animals were housed individually (polycarbonate cages, 33 × 16 × 13 cm; model 1144B, Tecniplast, Buguggiate, Italy) until the end of the experiment. The cages were placed in proximity to each other, allowing visual and auditory contact between animals, which helped to minimize isolation-induced stress. Following 1 week of post-operative recovery, the ovariectomized and sham-operated mice were divided into four experimental groups in a body mass-matched manner. Over the next 6 weeks, the ovariectomized (OVX-VWR, *n* = 10) and sham-operated (SHAM-VWR, *n* = 10) mice were subjected to voluntary wheel running, while the control groups (OVX-SED, *n* = 10 and SHAM-SED, *n* = 10) remained sedentary.

**Figure 1:**
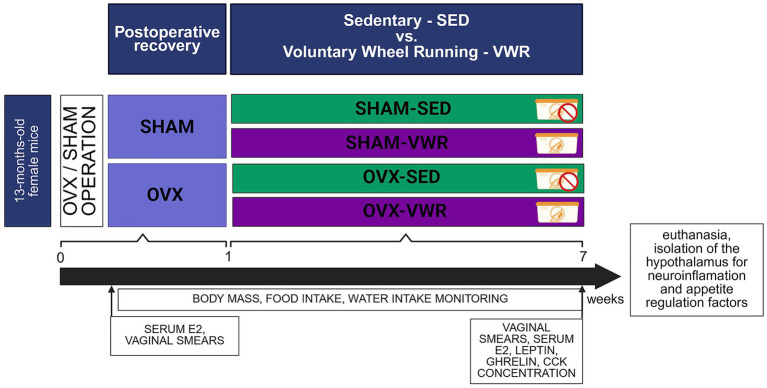
Scheme of experimental design. Serum cholecystokinin (CCK); serum 17-β-estradiol (E2); voluntary wheel running (VWR); sham operation (SHAM); bilateral ovariectomy (OVX); sedentary (SED). Created in BioRender. Malecki, A. (2025) https://BioRender.com/wyydj7u.

Throughout the seven-week experiment, all animals had *ad libitum* access to standard chow (Lobofeed B, Wytwórnia Pasz Morawski) (Supplementary Table S1.1) and drinking water. Body mass, food intake, and water consumption were measured weekly, starting from the day of the operation (body mass) or week post-operation (food and water intake). The estrus cycle stage and 17-*β*-estradiol (E2) concentration were assessed twice: once at the beginning of the experiment before operations, and again immediately before euthanasia at the end of the experiment. On the euthanasia day, mice were anesthetized and decapitated; then the hypothalamus and serum samples were collected. The experimental design is presented in Figure 1.

### 2.3 Surgical procedures and estrus cycle evaluation

Surgical procedures were conducted between 8:00 a.m. and 4:00 p.m. under aseptic conditions. Mice were fasted for 12 h before surgery. Blood samples were collected from the tail tip of all mice before operation, centrifuged (2000 x g, 10 min, 4 °C), and stored at −80 °C until further analysis. Bilateral ovariectomy was performed as described by Souza et al. (2019), with modifications. Briefly, the mice were anesthetized (IP injection: ketamine 100 mg/kg b.m., xylazine 10 mg/kg b.m.) and placed on a warming blanket. Small dorsolateral incisions were made through skin and muscle layers to expose the uterine horn, and the ovaries were carefully removed while leaving the uterus intact. The procedure was performed on the second flank. Control animals underwent the same surgical procedure (sham operation) but had their ovaries left intact. After surgery, the mice were housed individually for 1 week to facilitate post-operative recovery without intervention. All animals received paracetamol (24 mg/kg b.m. in drinking water) and gentamicin (IP injection: 80 mg/kg b.m.) for three consecutive days following the operations. Every effort was taken to monitor the animals for signs of distress or pain, as well as to observe wound healing.

The estrus cycle stages were assessed twice during the experiment: on the days of operations and euthanasia, directly after anesthesia. Vaginal lavages were performed by gently aspirating saline into the opening of the vaginal canal. The vaginal smears were stained with Giemsa solution (1 h, RT) and examined to identify estrus cycle-specific cell types (McLean et al., 2012) (Supplementary Table S2.1).

### 2.4 Voluntary wheel running

Two groups of female mice were subjected to voluntary wheel running: one group consisted of bilaterally ovariectomized (OVX-VWR) mice, while the other group included sham-operated (SHAM-VWR) mice. From the second week following operations, each mouse in both the SHAM-VWR and OVX-VWR groups was individually placed in cages equipped with running wheels (ACTI-PT2-MCR2, Actimetrics) and had continuous access to the wheels 24 h/day for the next 6 weeks. The mice in the sedentary control groups (OVX-SED and SHAM-SED) were housed in similar cages, but their wheels were immobilized. The distance run by each mouse was recorded individually using *ClockLab Data Collection* software and later analyzed with *ClockLab Analysis* (Actimetrics).

### 2.5 Euthanasia and tissue collection

Sample collection began at 9:00 a.m. Blood samples were collected from the tail tip of all mice before euthanasia and centrifuged (2000 x g, 10 min, 4 °C). Then all animals were anesthetized sequentially, with each mouse processed individually in a separate room (IP injection: ketamine 100 mg/kg/b.m., xylazine 10 mg/kg/b.m.). Following anesthesia, retro-orbital blood was collected and centrifuged (4,000 × g, 20 min, 4 °C). After blood sample collection, the anesthetized mouse was euthanized by decapitation. The brain was rapidly removed and placed in an Alto Stainless Steel 1 mm Mouse Brain Matrix (Coronal 40–75 gm, Roboz Surgical Instrument Co.). The fragment of the hypothalamus containing regions involved in appetite regulation was analyzed. The hypothalamus, intended for protein and RNA isolations, and serum samples for ELISA measurements were stored at −80 °C until further analysis. The uterus was isolated from all animals and weighed. In the case of sham-operated animals, the uteri were weighed after the ovaries were removed.

### 2.6 Western blotting

For protein extraction, hypothalamus tissue was sonicated in RIPA buffer containing protease and phosphatase inhibitors (20–188, Merck Millipore, 04693116001, Roche Holding AG, 4906845001, Roche Holding AG), and then centrifuged (12,000 x g, 20 min, 4 °C). Protein concentration was measured using the Pierce BCA Protein Assay Kit (23227, Thermo Fisher Scientific). Samples (20 μg protein) were separated on a gel (#4568086, Bio-Rad Laboratories, Inc.) and transferred (Trans-Blot® Turbo™ Transfer System, Bio-Rad Laboratories, Inc.). Membranes were blocked and then incubated with primary antibodies (overnight, 4 °C) (Supplementary Table S1.2). After washing in TBST, membranes were incubated with a secondary antibody (11–035-003, Jackson Immunoresearch Laboratories) and developed using Clarity™ Western ECL Substrate (#1705060, Bio-Rad Laboratories, Inc.). Chemiluminescent signals were detected with a ChemiDoc-Touch Imaging System (Bio-Rad Laboratories, Inc.). Original full-length gels and blots were included in Supplementary material 3. Protein band normalization was performed based on the total lane protein visualized under UV light, following Stain-Free Imaging Technology (Bio-Rad Laboratories, Inc.). The data were presented as the mean ± standard deviation (SD) of the fold change of the samples from the studied groups compared to the control SHAM-SED group.

### 2.7 RNA isolation, reverse transcription, and digital PCR

Total RNA from the hypothalamus was isolated using the miRNeasy Mini Kit (217004, Qiagen) and then reverse transcribed (1,000 ng of total RNA) with the Maxima H Minus First Strand cDNA Synthesis Kit, with dsDNase (K1682, Thermo Fisher Scientific), following the manufacturer’s protocols. Digital PCR (dPCR) was conducted using the QIAcuity EG PCR Kit (250111, Qiagen) along with target-specific primers (Genomed, Poland) (Supplementary Table S1.3) in the QIAcuity One 5 Plex system (Qiagen). The absolute number of gene copies was determined as the number of copies per 1 ng of isolated RNA. Normalization was performed according to the calculated endogenous control gene (*Gapdh*) normalization factors of SHAM-SED: 1; OVX-SED: 1.106; SHAM-VWR: 0.968; OVX-VWR: 1.095. Normalization factor values were calculated as fold change in gene copies per ng of RNA relative to the control SHAM-SED group (Millier et al., 2017). The data were presented as a mean ± SD of gene copies per 1 ng of RNA.

### 2.8 Serum 17-*β*-estradiol (E2), leptin, ghrelin, and cholecystokinin concentrations measurement

E2 concentration measurements in all animals were performed using serum samples obtained from blood collected from the mice tail tip on the days of operation and euthanasia. Leptin, ghrelin, and cholecystokinin concentrations were assessed in serum samples obtained from retro-orbital blood collected on the euthanasia day. E2, leptin, ghrelin, and cholecystokinin levels were determined using commercially available ELISA kits (E2: ab108667, Abcam; Ghrelin: EIA-GHR-1, RayBiotech; Cholecystokinin: EIA-CCK, RayBiotech; Leptin: ab100718, Abcam) following the manufacturer’s instructions.

### 2.9 Statistical analysis

Prism 10.1.2 (GraphPad Software) was used for statistical analyses and figure generation. Results are presented as means ± SD. The normality of each dataset was assessed using the Shapiro–Wilk test. Upon confirming that the data followed a normal distribution, a t-test with Welch correction was employed to determine significant differences between the two groups (uterus mass). To compare the initial to the final body mass within a group, a paired t-test was conducted. An ordinary one-way ANOVA was applied to evaluate differences among four experimental groups (initial and final body mass, weight gain, average food intake, feeding efficiency ratio (FER%), and water intake). Additionally, a repeated measures (RM) one-way ANOVA with Tukey’s multiple comparisons test was performed to analyze body mass data collected at different time points within one group throughout the experimental period. A two-way ANOVA with Tukey’s multiple comparison tests was used to assess the main effects of operation, voluntary wheel running, and the interaction between these factors (Western blotting, dPCR, and ELISA). For data that did not conform to a normal distribution, the Mann–Whitney test (distance covered in 1 week between two groups), and the Friedman test with Dunn’s multiple comparisons test (distance covered during 6 weeks of voluntary wheel running within one group) were employed. In all analyses, *p*-values of less than 0.05 were deemed statistically significant. The results of the statistical analyses are included in Supplementary material 2.

## 3 Results

### 3.1 Reduced running activity was sufficient to stabilize the body mass increase in the middle-aged mice following ovariectomy

**Figure 2:**
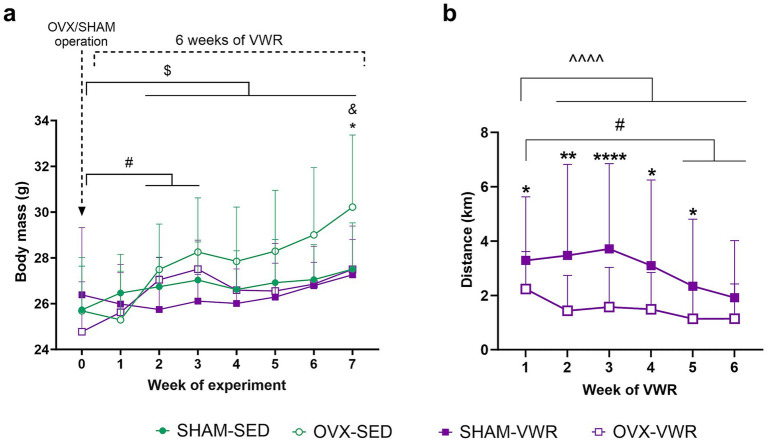
Reduced running activity was sufficient to stabilize the body mass increase in the middle-aged mice following ovariectomy. Ovariectomy led to a gradual increase in body mass of middle-aged female mice (OVX-SED: Week 2–7 vs. Week 0: ^$^*p* < 0.05, OVX-VWR: Week 2–3 vs. Week 0: ^#^*p* < 0.05). After 7 weeks of experiment, the final body mass of ovariectomized sedentary mice was higher than sham-operated controls (SHAM-SED vs. OVX-SED: ^&^*p* < 0.05) and the operation-matched running mice (OVX-SED vs. OVX-VWR: *p < 0.05) **(a)**. During the first 5 weeks of voluntary wheel running (VWR), ovariectomy reduced covered running distances (SHAM-VWR vs. OVX-VWR: *p < 0.05, **p < 0.01, ****p < 0.0001). Sham-operated mice covered shorter distances in the 5th and 6th weeks compared to the 1st week (SHAM-VWR: Week 5, 6 vs. Week 1: #*p* < 0.05). Conversely, ovariectomized mice ran longer distances in the 1st week than in subsequent weeks (OVX-VWR: Week 2–6 vs. Week 1: ^^^^*p* < 0.0001) **(b)**. Values are presented as mean ± SD. Ordinary/RM one-way ANOVA followed by Tukey’s multiple comparison test (Body mass), Friedman test with Dunn’s multiple comparisons test (Distance), and Mann–Whitney test (Distance) were performed. SHAM - sham operation, OVX, bilateral ovariectomy; SED, sedentary; VWR, voluntary wheel running. *n* = 10 per group.

At the beginning of the experiment, the initial body mass of animals did not differ between groups. Throughout the experimental period, the body mass of the ovariectomized sedentary animals gradually increased from the second week of the experiment, compared to their initial body mass. In contrast, the running mice that underwent ovariectomy exhibited increased body mass after the second and third weeks, after which their body mass stabilized (Figure 2a). After 7 weeks of the experiment, the final body mass of the animals in the OVX-SED (*p* < 0.0001) group increased. Notably, the final body mass of the ovariectomized sedentary mice (OVX-SED) was higher than that of the sham-operated sedentary (SHAM-SED, *p* = 0.0498) and operation-matched running (OVX-VWR, *p* = 0.0495) animals (Figure 2a). Moreover, ovariectomized sedentary mice gained weight most excessively (SHAM-SED vs. OVX-SED: *p* = 0.0274) and exhibited the highest feeding efficiency ratio (FER%) (SHAM-SED vs. OVX-SED: *p* = 0.0217) (Table 1). In sham-operated mice, voluntary wheel running increased total average daily food intake compared to sedentary mice (SHAM-SED vs. SHAM-VWR: *p* = 0.0039) (Table 1). Neither ovariectomy nor voluntary wheel running had a significant effect on total average daily water consumption (Table 1).

**Table 1:** Characteristics of body mass, food, and water intake of all mice.

Parameter	SHAM-SED	OVX-SED	SHAM-VWR	OVX-VWR	*p*-value
Weight gain (g/day)	0.036 ± 0.03	0.093 ± 0.03*	0.018 ± 0.05	0.056 ± 0.05	0.0030
Food intake (g/day)	4.42 ± 0.22	4.69 ± 0.29	5.04 ± 0.50**	4.81 ± 0.41	0.0071
FER (%)	0.81 ± 0.672	1.96 ± 0.57*	0.40 ± 0.98	1.16 ± 1.06	0.0017
Water intake (ml/day)	7.81 ± 1.62	6.54 ± 1.32	9.19 ± 2.90	7.23 ± 2.65	0.0702

Values are presented as mean ± SD. Feeding efficiency ratio (FER, %) was calculated as: (Weight gain/Food intake) *100. One-way ANOVA followed by Tukey’s multiple comparisons test: SHAM-SED vs. *p < 0.05, ** *p* < 0.01. SHAM, sham operation; OVX, bilateral ovariectomy; SED, sedentary; VWR, voluntary wheel running. *n* = 10 per group.

Starting from the fifth week of voluntary wheel running, the sham-operated mice began to adapt, resulting in a lowered running distance compared to earlier weeks (1st vs. 2nd–6th week: *p* < 0.0001). In contrast, the ovariectomized mice covered a longer distance in the first week than in the following 5 weeks of voluntary wheel running (1st vs. 2nd–6th week: *p* < 0.0001) (Figure 2b). Ovariectomy resulted in shorter distance covered in 1st to 5th weeks (1st week: *p* = 0.0131; 2nd week: *p* = 0.0012; 3rd week: *p* < 0.0001; 4th week: *p* = 0.0104; 5th week: *p* = 0.0389) (Table 2). The covered mean weekly distance between sham-operated and ovariectomized did not differ in the 6th week.

**Table 2:** The average daily distance (km) covered in the active phase, in every week, of 6 weeks of voluntary wheel running.

Group	1 week	2 week	3 week	4 week	5 week	6 week
SHAM-VWR	3.29 ± 2.35	3.47 ± 3.36	3.72 ± 3.13	3.1 ± 3.16	2.34 ± 2.47	1.92 ± 2.1
OVX-VWR	2.24 ± 1.37	1.44 ± 1.3	1.57 ± 1.46	1.49 ± 1.36	1.14 ± 1.09	1.14 ± 1.28
*p*-value	0.0131	0.0012	< 0.0001	0.0104	0.0389	0.1266

Results of the Mann–Whitney test of the average weekly distance compared between the two running groups. Values are presented as mean ± SD (km). SHAM, sham operation; OVX, bilateral ovariectomy; SED, sedentary; VWR, voluntary wheel running. *n* = 6–7 per group.

### 3.2 Voluntary wheel running changed the hypothalamic ERβ expression level

**Figure 3:**
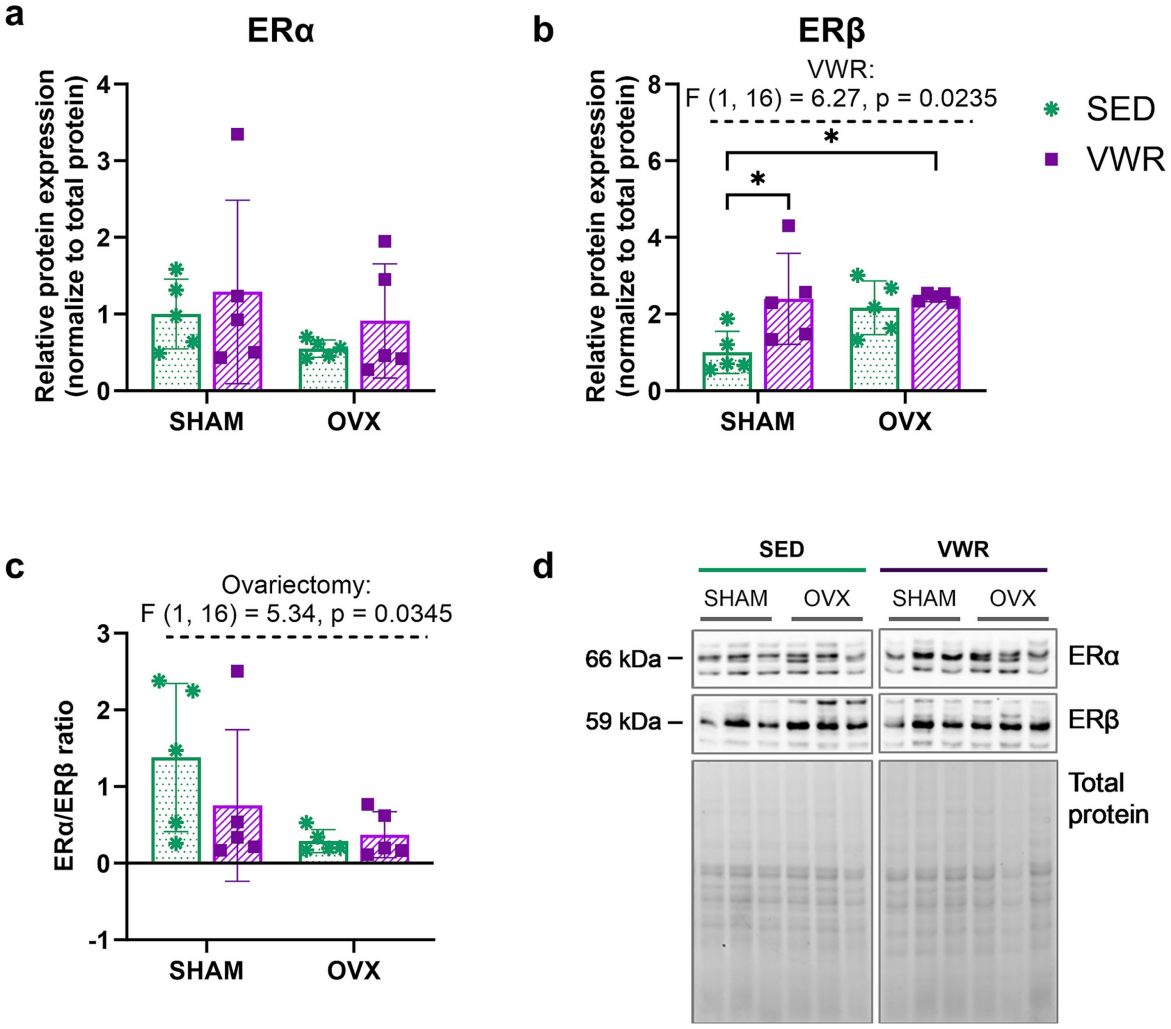
Effects of 6 weeks of voluntary wheel running on estrogen receptor (ER) expression in the hypothalamus of middle-aged (13-month-old) mice, 7 weeks after bilateral ovariectomy or sham operation **(a,b)**. The ERα to ERβ expression level ratio was reduced following ovariectomy **(c)**. The running ovariectomized and sham-operated mice exhibited higher ERβ expression compared to sedentary animals that underwent sham operation. Representative images of Western blotting analysis, cropped bands from different parts of the same gel, and blots are shown **(d)**. The samples were derived from the same experiment, and gels/blots were processed in parallel. Values are presented as mean ± SD. Two-way ANOVA followed by Tukey’s multiple comparison test was performed. *p < 0.05. SHAM, sham operation; OVX, bilateral ovariectomy; SED, sedentary; VWR, voluntary wheel running. *n* = 5 per group.

Even though serum E2 levels remained unchanged, all ovariectomized mice had a lower uterine mass compared to sham-operated animals (Table 3). Additionally, all ovariectomized mice were in the metestrus/diestrus stage, indicating the cessation of the ovarian cycle (Supplementary Table S2.1). The hypothalamic estrogen receptor (ER) *α* expression level did not change significantly, whereas the ERα to ERβ expression ratio was significantly reduced through ovariectomy (*p* = 0.0345) (Figures 3a,c). The voluntary wheel running increased the hypothalamic ERβ expression level, with significant differences between running groups and sedentary sham-operated mice (SHAM-SED vs. SHAM-VWR: *p* = 0.0409, SHAM-SED vs. OVX-VWR: *p* = 0.0350) (Figures 3b,d).

**Table 3:** Average serum 17-β-estradiol concentration and average uterine mass of middle-aged animals 7 weeks after operations.

Parameter	SHAM	OVX	*p*-value
17-β-estradiol (pg/ml) – Week 0	32.87 ± 7.56	32.12 ± 11.65	0.8089
17-β-estradiol (pg/ml) – Week 7	34.30 ± 9.91	31.22 ± 12.57	0.3964
Uterus mass (mg)	125.2 ± 46.50	23.92 ± 5.21	< 0.0001

Values are presented as mean ± SD. Unpaired t-test with Welch’s correction: SHAM vs. OVX. SHAM, sham operation; OVX, bilateral ovariectomy. *n* = 20 per group.

### 3.3 Voluntary wheel running modulated the expression level of appetite-related factors in the hypothalamus of middle-aged mice in accordance with ovarian status

**Figure 4:**
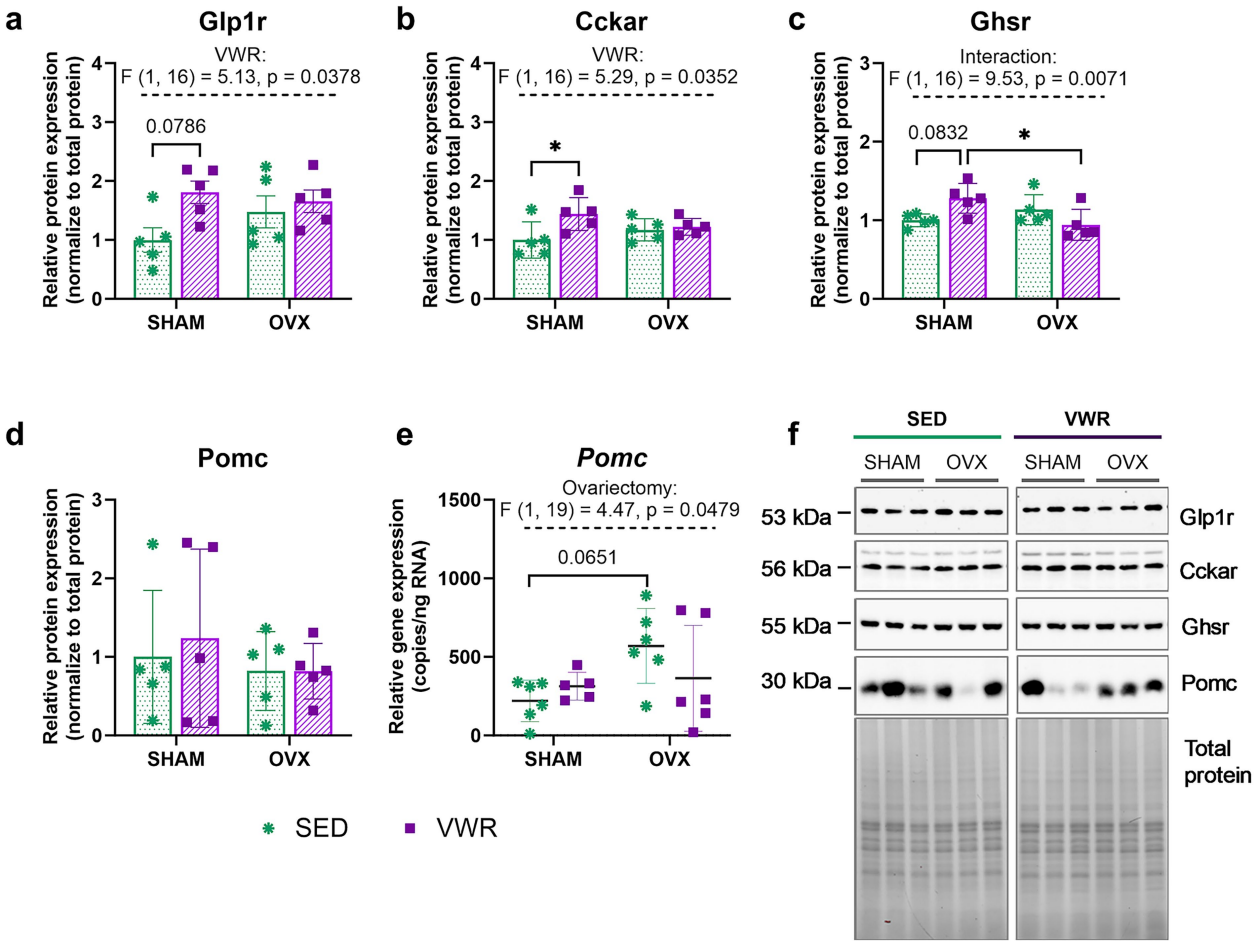
Effects of 6 weeks of voluntary wheel running on Glp1r **(a)**, Cckar **(b)**, Ghsr **(c)**, Pomc **(d)** proteins, and *Pomc* gene **(e)** expression in the hypothalamus of middle-aged (13-month-old) mice, 7 weeks after bilateral ovariectomy or sham operation. Among sham-operated mice, voluntary wheel running increased Cckar and Ghsr expression. Representative images of Western blotting analysis, cropped bands from different parts of the same gel, and blots are shown **(f)**. The samples were derived from the same experiment, and gels/blots were processed in parallel. Values are presented as mean ± SD. Two-way ANOVA followed by Tukey’s multiple comparison test was performed. *p < 0.05. SHAM, sham operation; OVX, bilateral ovariectomy; SED, sedentary; VWR, voluntary wheel running. *n* per group: dPCR (5–6), Western blot (5).

Six weeks of wheel running increased cholecystokinin receptor (Cckar) expression levels in the hypothalamus, with significant differences observed between the sham-operated sedentary and sham-operated voluntary wheel running groups (*p* = 0.0444) (Figures 4b,f). The serum concentrations of ghrelin and cholecystokinin remained unchanged (Table 4). Among the running animals, the OVX mice exhibited lower levels of ghrelin receptor (Ghsr) expression compared to those that underwent sham operation (*p* = 0.0393) (Figure 4c). Voluntary running in sham-operated mice tended to increase glucagon-like peptide-1 receptor (Glp1r) and Ghsr expression levels (SHAM-SED vs. SHAM-VWR: Glp1r: *p* = 0.0786, Ghsr: *p* = 0.0832) (Figures 4a,c). While proopiomelanocortin (Pomc) protein expression remained unchanged (Figure 4d), the main effect of the operation (SHAM vs. OVX) on *Pomc* gene expression (*p* = 0.0479) was noted.

**Table 4:** Effects of 6 weeks of voluntary wheel running on serum ghrelin and cholecystokinin concentration in middle-aged mice, 7 weeks after bilateral ovariectomy or sham operation.

Parameter	SHAM-SED	OVX-SED	SHAM-VWR	OVX-VWR
Ghrelin (ng/ml)	27.14 ± 14.83	27.46 ± 23.01	28.37 ± 13.07	33.01 ± 27.82
Cholecystokinin (pg/ml)	264.3 ± 65.12	288.1 ± 69.63	242.4 ± 34.92	293.5 ± 116.2

Values are presented as mean ± SD. SHAM, sham operation; OVX, bilateral ovariectomy; SED, sedentary; VWR, voluntary wheel running. *n* = 6–7 per group.

OVX mice showed a tendency to increase in *Pomc* gene expression (SHAM-SED vs. OVX-SED: *p* = 0.0651) (Figure 4e). The gene expression levels of agouti-related protein (Agrp), cocaine and amphetamine regulated transcript (Cart), and neuropeptide Y (Npy) remained unchanged between groups (Table 5).

**Table 5:** Effects of 6 weeks of voluntary wheel running on gene expression levels in the hypothalamus of middle-aged mice, 7 weeks after bilateral ovariectomy or sham operation.

Gene	SHAM-SED	OVX-SED	SHAM-VWR	OVX-VWR
*Cart*	864.3 ± 228.3	1,113 ± 474.2	786.0 ± 171.4	990.9 ± 610.2
*Agrp*	14.26 ± 8.00	15.81 ± 6.08	15.32 ± 4.04	13.75 ± 6.46
*Npy*	661.7 ± 345.7	818.3 ± 346.8	928.5 ± 393.4	783.6 ± 386.3
*Nlrp3*	3.87 ± 2.18	4.10 ± 1.39	4.57 ± 2.02	3.48 ± 1.12
*Rela*	37.69 ± 15.83	33.83 ± 13.07	39.72 ± 5.53	32.56 ± 13.23
*Relb*	6.21 ± 3.26	5.96 ± 0.84	6.65 ± 1.47	4.69 ± 2.30
*Il-6*	1.07 ± 0.82	1.22 ± 0.40	1.31 ± 0.70	1.04 ± 0.67
*Tnf*	0.54 ± 0.45	0.39 ± 0.25	0.47 ± 0.13	0.93 ± 0.63

Values are presented as mean ± SD of gene copies per ng of total RNA. Two-way ANOVA followed by Tukey’s multiple comparison test was performed. SHAM, sham operation; OVX, bilateral ovariectomy; SED, sedentary, VWR, voluntary wheel running. *n* = 5–7 per group.

**Figure 5:**
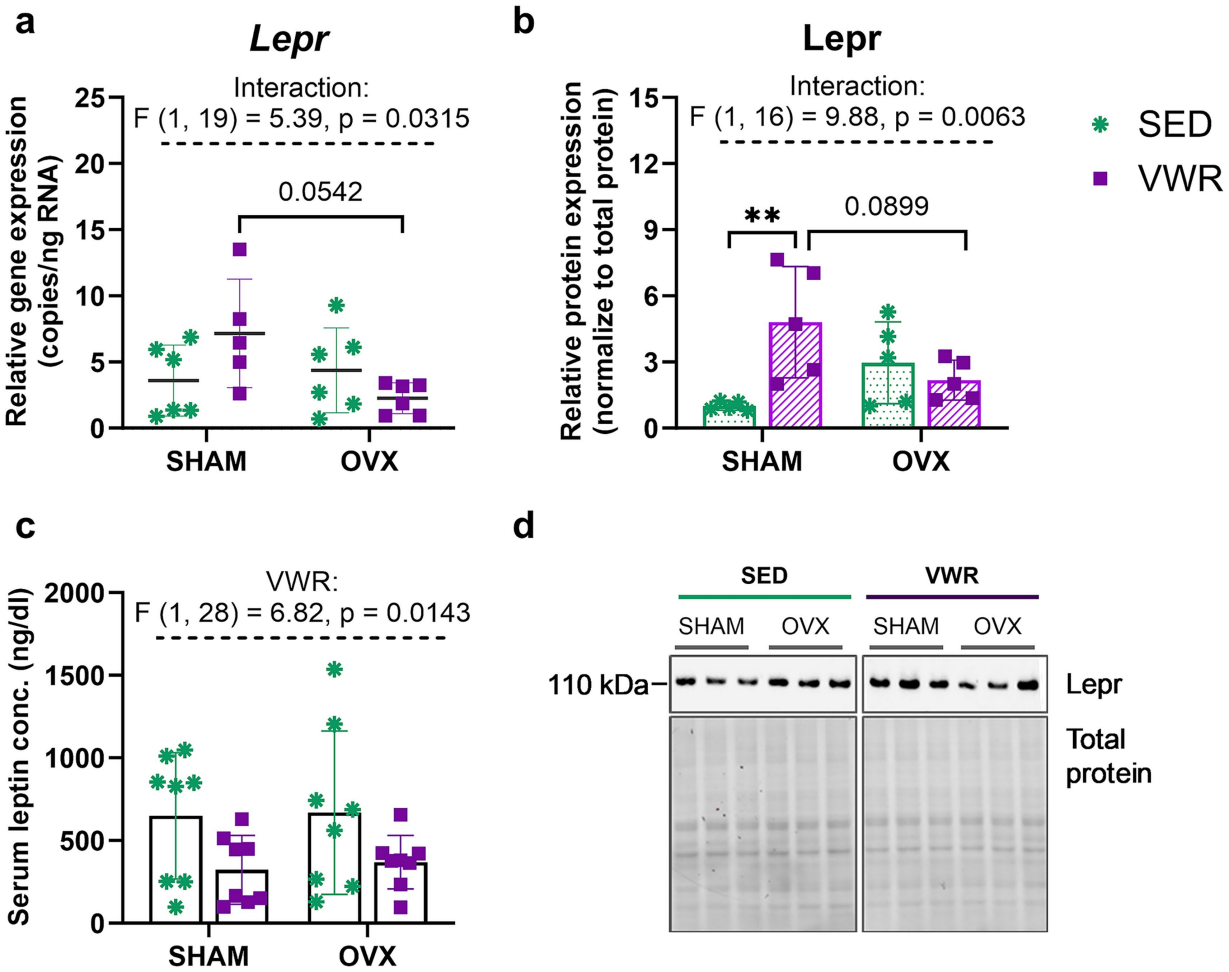
Effects of 6 weeks of voluntary wheel running on leptin receptor (Lepr) gene **(a)** and protein **(b)** expression levels in the hypothalamus of middle-aged (13-month-old) mice, 7 weeks after bilateral ovariectomy or sham operation. Among sham-operated mice, voluntary wheel running increased Lepr protein expression. After 7 weeks of experiment, no changes between groups were noted in the serum leptin concentration **(c)**. Representative images of Western blotting analysis, cropped bands from different parts of the same gel, and blots are shown **(d)**. The samples were derived from the same experiment, and gels/blots were processed in parallel. Values are presented as mean ± SD. Two-way ANOVA followed by Tukey’s multiple comparison test was performed. **p < 0.01. SHAM, sham operation; OVX, bilateral ovariectomy; SED, sedentary; VWR, voluntary wheel running. *n* per group: dPCR (5–6), Western blot (5), ELISA (8).

The voluntary running increased leptin receptor (Lepr) expression level in sham-operated mice (SHAM-SED vs. SHAM-VWR: *p* = 0.0097), but not in ovariectomized mice (Figures 5b,d). Two-way ANOVA revealed a significant interaction of operation and running on the expression of the *Lepr* gene (*p* = 0.0315). Although no significant changes between groups were revealed after 6 weeks of wheel running, *Lepr* gene expression tended to be lower in ovariectomized mice than in sham-operated animals (SHAM-VWR vs. OVX-VWR: *p* = 0.0542) (Figure 5a). Also, a similar pattern of change between SHAM-VWR and OVX-VWR groups was observed in Lepr protein expression (*p* = 0.0899) (Figure 5b). Running significantly reduced the serum leptin concentrations (*p* = 0.0143). However, the experimental groups had no differences in the studied hormone concentration (Figure 5c).

### 3.4 Voluntary running altered hypothalamic neuroinflammation regardless of ovarian status

**Figure 6:**
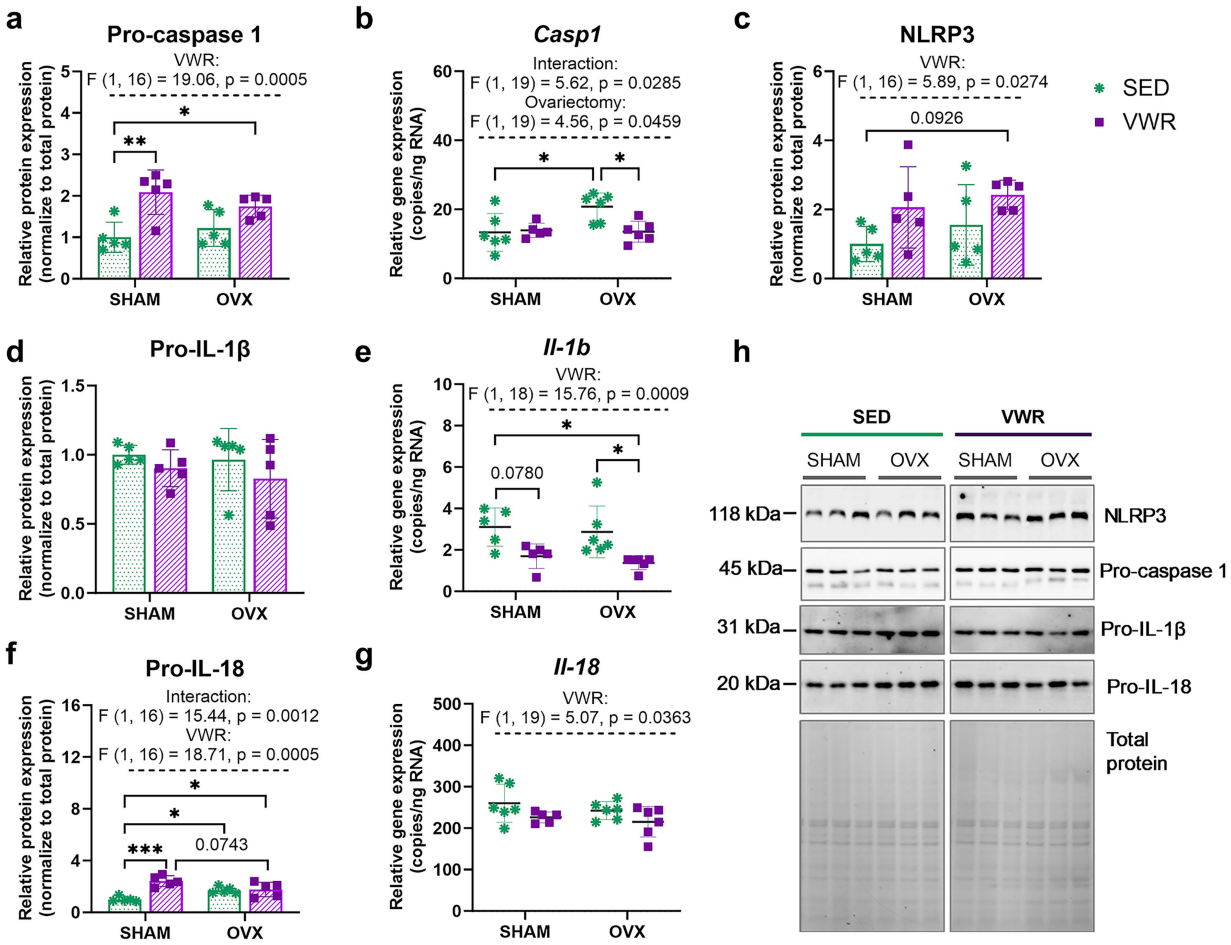
Effects of 6 weeks of voluntary wheel running on expression levels of the NLRP3 inflammasome components in the hypothalamus of middle-aged (13-month-old) mice, 7 weeks after bilateral ovariectomy or sham operation **(a–g)**. The running ovariectomized and sham-operated mice exhibited higher pro-caspase 1 and pro-IL-18 expression levels than sedentary animals that underwent sham operation. Representative images of Western blotting analysis, cropped bands from different parts of the same gel, and blots are shown **(h)**. The samples were derived from the same experiment, and gels/blots were processed in parallel. Values are presented as mean ± SD. Two-way ANOVA followed by Tukey’s multiple comparison test was performed. *p < 0.05, **p < 0.01, ***p < 0.001. SHAM, sham operation; OVX, bilateral ovariectomy; SED, sedentary; VWR, voluntary wheel running. n per group: dPCR (5–6), Western blot (5).

Ovariectomy upregulated *Casp1* gene expression in the sedentary mice (*p* = 0.0195). This effect was significantly diminished after voluntary wheel running (OVX-SED vs. OVX-VWR: *p* = 0.0230) (Figure 6a). The voluntary running decreased expression of the *Il-1b* in OVX mice (OVX-SED vs. OVX-VWR: *p* = 0.0323, SHAM-SED vs. OVX-VWR: *p* = 0.0171), and induced downregulation in SHAM mice, but without reaching significance level (SHAM-SED vs. SHAM-VWR: *p* = 0.0780) (Figure 6d). Additionally, a reduction in *Il-18* gene expression was observed in running mice (*p* = 0.0363) (Figure 6f).

The main effect of voluntary running on NLRP3 (*p* = 0.0274), with no significant changes between groups, was revealed. The wheel running tended to upregulate NLRP3 expression in ovariectomized mice, as compared to the sham-operated group (*p* = 0.0926) (Figures 6c,e). In all running animals, regardless of ovarian status, the expression level of pro-caspase 1 was increased when compared to the SHAM-SED group (SHAM-SED vs. SHAM-VWR: *p* = 0.0037, SHAM-SED vs. OVX-VWR: *p* = 0.0498) (Figure 6b). In sedentary animals, OVX mice had an increased level of pro-interleukin (IL)-18 compared to SHAM mice (*p* = 0.0459). After 6 weeks of Voluntary running, mice exhibited higher levels of pro-IL-18 expression than the controls (sham-operated sedentary animals) (SHAM-SED vs. SHAM-VWR: *p* = 0.0001, SHAM-SED vs. OVX-VWR: *p* = 0.0265) (Figure 6g).

**Figure 7:**
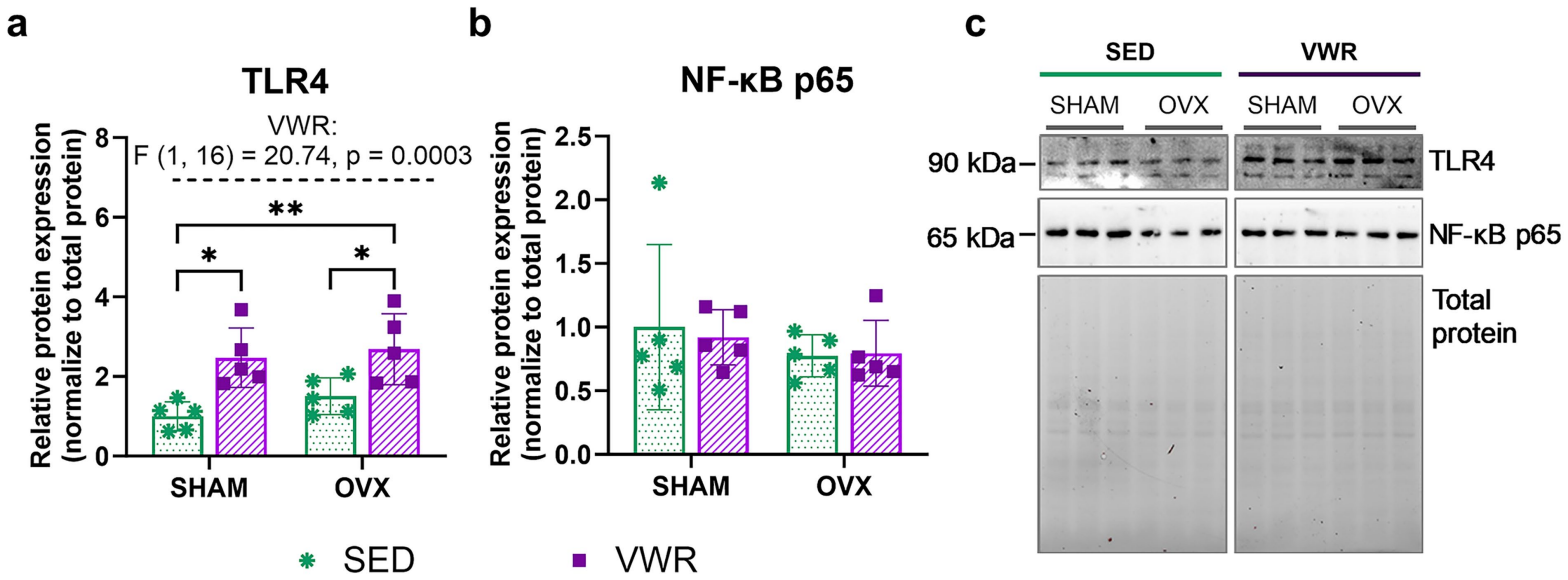
Effects of 6 weeks of voluntary wheel running on expression levels of TLR4 **(a)**, and NF-κB p65 **(b)** in the hypothalamus of middle-aged (13-month-old) mice, 7 weeks after bilateral ovariectomy or sham operation. The wheel running upregulated TLR4 expression levels in ovariectomized and sham-operated mice. Representative images of Western blotting analysis, cropped bands from different parts of the same gel, and blots are shown **(c)**. The samples were derived from the same experiment, and gels/blots were processed in parallel. Values are presented as mean ± SD. Two-way ANOVA followed by Tukey’s multiple comparison test was performed. *p < 0.05, **p < 0.01. SHAM, sham operation; OVX, bilateral ovariectomy; SED, sedentary; VWR, voluntary wheel running. *n* = 5 per group.

The combination of running and ovariectomy induced an increase in the hypothalamic expression level of toll-like receptor 4 (TLR4) as compared to both sedentary groups (SHAM-SED vs. OVX-VWR: *p* = 0.0042; OVX-SED vs. OVX-VWR: *p* = 0.0493). Voluntary running mice exhibited a higher level of TLR4 expression than the sedentary animals (*p* = 0.0122) (Figures 7a,c). No changes in pro-IL-1β (Figure 6e) and nuclear factor kappa-light-chain-enhancer of activated B cells (NF-κB) p65 (Figure 7b) expression levels were noted. No significant differences were observed between groups after 7 weeks of voluntary running in gene expression levels of *Nlrp3, Il-18*, *Rela*, *Relb, Il-6*, and *Tnf* (Table 5).

## 4 Discussion

In this study, we showed that ovariectomy in middle-aged mice significantly affected their body weight. However, these changes were lessened by the introduction of voluntary wheel running. Additionally, this intervention modified central appetite-regulating factors potentially through ERs-dependent signaling. ERs in the hypothalamus play distinct but interconnected roles in regulating energy homeostasis and appetite. Activation of ERα has been consistently shown to have strong anorexigenic effects, as studies have indicated that ovariectomized adult rats experience reduced food intake and decreased body mass following ERα activation (Santollo et al., 2007). Moreover, ovariectomy may induce hyperphagia and fat accumulation, which were reversed by E2 treatment, and this effect may be diminished by ERβ inhibition (Liang et al., 2002). Studies indicate that ovariectomy may increase gene and protein expression of both ERα and ERβ in the hypothalamic preoptic area (POA), which was accompanied by a decrease in circulating E2 levels (Ma et al., 2011). Conversely, other research indicated that ovariectomy decreased the expression of ERα and ERβ in the same brain area (Yang et al., 2022). In the present study, although in middle-aged mice we did not observe changes in the expression levels of hypothalamic ERα and ERβ after ovariectomy, the ratio of ERα to ERβ decreased. It is worth noting that an age-related decline in hypothalamic ERβ cell number was previously demonstrated to start from middle age, in contrast to adult rodents (Chakraborty et al., 2003). Additionally, the expression of ERs can change over time following ovariectomy (Baek et al., 2024). This observation may support our current findings, which indicate changes in the ERα/ERβ ratio after ovariectomy. Here, in conjunction with the decreased ERα/ERβ ratio, the ovariectomized sedentary mice gained weight excessively, with almost 20% higher body mass than their initial body mass, which may be understood as the initiation of the onset of overweight. Previous studies have shown that ERβ activation enhances leptin sensitivity and suppresses ghrelin-induced orexigenic signaling, thereby contributing to the maintenance of energy homeostasis (Ropelle et al., 2021). The observed changes in ERβ expression in our study may therefore influence the balance between anorexigenic and orexigenic neuronal activity. Moreover, since postmenopausal estrogen decline is associated with leptin resistance and increased ghrelin signaling, our findings suggest that physical activity could partially restore ERβ-mediated modulation of these pathways, supporting its beneficial role in appetite control and metabolic regulation during the postmenopausal period. Importantly, the ovariectomized sedentary mice did not consume more food than sham-operated animals. However, the observed increase in the feeding efficiency ratio suggests a shift toward more efficient energy storage and reduced metabolic output. This observation aligns with research indicating that ovariectomy contributes to increased adiposity and weight gain, even in the absence of significant changes in food intake (Rogers et al., 2009). On the contrary, we noted that running ovariectomized mice did not exhibit signs of being overweight, increased food consumption, or disruption of the feeding efficiency ratio. Previous research has shown that voluntary exercise decreases weight gain and fat accumulation in ovariectomized mice by decreasing high-fat food consumption, likely due to increased energy expenditure and enhanced metabolic efficiency (Cordeira and Monahan, 2019). In our research, in sham-operated mice, voluntary wheel running increased food consumption, without changes in body mass. A previous study confirmed that despite increased food intake after running, the mice-maintained energy balance (Styner et al., 2014). This may indicate that the energy expended through exercise offsets the excess calories consumed, thereby maintaining overall energy balance. As we previously demonstrated, ovariectomy reduced running activity, including total average daily running distance, average running speed, and distance covered during the dark phase, compared with sham-operated animals (Grabowska et al., 2025). In the present study, voluntary wheel running in sham-operated mice (characterized by higher running intensity) and in ovariectomized mice (with lower running intensity) both resulted in increased ERβ expression, suggesting that even moderate physical activity may exert beneficial regulatory effects on ER signaling. This further supports the observation regarding the role of ERβ’s in the regulation of energy metabolism by physical activity (Kaidah et al., 2016; Zuloaga et al., 2014). Additionally, Grigsby et al. suggested that ERα activation in the medial preoptic area (MPOA) increases wheel running behavior, while ERβ activation may inhibit ERα signaling. Moreover, rats with naturally low voluntary running behavior exhibited higher MPOA ERs gene expression, lower uterus mass, and lower E2 circulating levels compared to wild-type animals, suggesting their low running may be due to E2 drop (Grigsby et al., 2020). Here, we observed lowered uterine weight after ovariectomy; however, we have not noted changes in E2 serum concentration between sham-operated and ovariectomized mice. Thus, as we observed that running ovariectomized mice had a lowered ERα/ERβ ratio, together with increased ERβ level, we could speculate that running behavior is strongly associated with the hypothalamic ERs expression rather than E2 circulating levels, especially in middle-aged females.

Several studies have shown that the ovariectomy-induced rise in leptin secretion (Burch et al., 2022; Chen and Heiman, 2001; Jackson et al., 2011; Chu et al., 1999; Isken et al., 2008) has been associated with increased body mass (Burch et al., 2022; Chu et al., 1999; Isken et al., 2008), potentially due to decreased sensitivity to leptin signaling (Chu et al., 1999). Following ovariectomy, the leptin level may proportionally increase with body mass, while the Lepr level decreases (Burch et al., 2022; Ivić et al., 2016). Also, ovariectomy has been found to lead to a transient increase in circulating ghrelin levels, which may correlate with the occurrence of hyperphagia and overweight (Clegg et al., 2007). Previously, no changes in serum ghrelin levels or hypothalamic Ghsr levels were observed after ovariectomy, despite an increase in body mass (Burch et al., 2022). In our study, we found that ovariectomy in middle-aged mice induced weight gain, which was not associated with changes in serum leptin and ghrelin levels, as well as Ghsr, and Lepr protein and gene expression levels in the hypothalamus. Similarly, our results align with those of other research, which demonstrated no significant differences in *Lepr* gene expression levels or food intake associated with higher body mass, as well as reduced spontaneous activity after ovariectomy. However, unlike our findings, they demonstrated increased circulating leptin concentration (Isken et al., 2008). We observed that running decreased serum leptin levels in all mice regardless of the operation, while ghrelin serum concentration remained unchanged. Moreover, voluntary wheel running led to an increase in Lepr and Ghsr levels only in sham-operated, but not ovariectomized, middle-aged mice. Similar results have been observed in studies involving adult and middle-aged rodents, concerning circulating leptin concentration (Kimura et al., 2004; Wang et al., 2025; Zhao et al., 2011; Benatti et al., 2008) and Lepr levels in the hypothalamus (Ruegsegger et al., 2016) after exercise training. It has been previously shown that exercise causes a significant increase in circulating ghrelin concentration (Ghanbari-Niaki et al., 2009; Fathi et al., 2010) or hypothalamic ghrelin level, together with no changes in circulating ghrelin and hypothalamic Ghsr expression (Wang et al., 2008). Since ghrelin is a potent orexigenic peptide (Singh et al., 2023), the increased expression of Ghsr in the sham-operated group may indicate that exercise induced CNS promotion of hunger signaling, which could be confirmed by the observation that those animals consumed more food. However, we showed that among running mice, ovariectomy resulted in a significant reduction in Ghsr levels. Despite differential responses to voluntary running between sham-operated and ovariectomized mice in terms of Lepr and Ghsr levels, both groups developed similar phenotypes and peripheral effects, including reduced body mass and leptin concentration. Leptin and ghrelin receptors are frequently co-localized with ERs within the brain, suggesting their interconnected role with estrogen signaling in appetite regulation (Dubern and Clement, 2012; Gao and Horvath, 2008; Frazao et al., 2014). Here, we observed that ovariectomy disrupted the ERs ratio. Thus, we could hypothesize that preservation of ovarian function may be necessary for exercise-induced improvements in leptin and ghrelin CNS signaling.

Here, we have not observed changes in serum cholecystokinin concentrations following either ovariectomy or voluntary wheel running. Moreover, ovariectomy has not affected the hypothalamic Cckar and Glp1r levels, whose activation may lead to an appetite suppression (Wank et al., 1994; Park et al., 2019). Surprisingly, the voluntary wheel running enhanced the expression of Cckar and Glp1r proteins in sham-operated, but not in ovariectomized mice. This finding contrasts with Ghsr changes after voluntary running in sham-operated mice, which were consistent with the promotion of food consumption. Previously, the Glp1r expression level upregulation in the hypothalamus was also demonstrated after the high-intensity resistance exercise training (Park et al., 2019). Considering the above and the observation that sham-operated mice exhibited higher running activity compared to ovariectomized mice, the intensity of physical activity may be a limiting factor in its effect on Cckar and Glp1r levels.

In our study, we found that ovariectomy in sedentary mice induced an upregulation of *Pomc* gene expression in the hypothalamus, without altering Pomc protein levels. The discrepancies observed between mRNA and protein levels may result from post-transcriptional regulatory mechanisms or dilution effects due to the analysis of the whole hypothalamus rather than discrete nuclei such as the ARC, paraventricular nucleus (PVN), or MPOA. Previous research has indicated that ovariectomy decreased the activation of POMC neurons, impairing their ability to inhibit NPY/AgRP neurons (Stincic et al., 2018). Additionally, the inhibition of ERα activation may promote NPY neurons activation, potentially impairing the estrogen-mediated decrease in food intake, weight gain, and increase in energy expenditure (Fernandois et al., 2024; Ye et al., 2024). Also, it has been previously reported that ovariectomy may reduce the expression of *Cart* in the hypothalamus without affecting food consumption, despite leading to an increase in body weight (Isken et al., 2008). We did not observe differences in the expression of the *Cart*, *Agrp*, and *Npy* genes following ovariectomy or voluntary wheel running. Consistent with our findings, high-intensity exercise has not altered *Pomc* and *Npy* gene expression levels; however, unlike our study, the researchers did not record any changes in food intake (Khajehnasiri et al., 2019). Other studies have demonstrated that moderate-intensity exercise activates NPY/AgRP neurons in the ARC without affecting POMC neurons, which was associated with increased food intake (Landry et al., 2022; Bunner et al., 2020). While the mentioned studies focus on changes in neuronal activity, we measured gene expression levels, which do not always correlate with protein data. This has been assumed as one of our limitations. Moreover, we conducted measurements using the whole hypothalamus without isolating specific areas. This approach may distort the perception of subtle differences specific to individual areas of the hypothalamus, which play distinct roles in various physiological functions.

Several studies on ovariectomized animals suggest that a lack of ovarian function may induce proinflammatory response within the brain (Saad et al., 2022; Mouihate, 2014). Additionally, the onset of neuroinflammation associated with disruption of ER signaling has also been observed during metabolic dysfunction (Baek et al., 2024; Benedusi et al., 2017; Ahmed and Hassanein, 2012). Increased expression of the Npy has been linked to metabolic alterations in ovariectomized mice, but also exacerbated neuroinflammation (Oberto et al., 2022). As we observed a differential response of appetite-regulating factors to voluntary wheel running between sham-operated and ovariectomized middle-aged mice, we investigated whether these discrepancies were associated with neuroinflammatory changes. Previous studies have indicated that ovariectomy may activate microglia, as well as increase hypothalamic IL-1β levels, together with alterations in ERs expression (Baek et al., 2024). Although we noted changes in the ERs ratio following ovariectomy, we did not find differences in the protein and gene levels of IL-1β. We showed that ovariectomy-induced upregulation of the *Casp1* gene in sedentary mice was diminished in running mice. We demonstrated that among proteins involved in the inflammatory response, voluntary wheel running upregulated the levels of pro-caspase 1, pro-IL-18, and TLR4 in both sham-operated and ovariectomized mice. Notably, ovariectomy alone also increased the pro-IL-18 level. However, sham-operated animals exhibited higher pro-IL-18 levels than those ovariectomized, suggesting that the response to physical activity is more pronounced in intact animals. The observed increase in pro-IL-18 and pro-caspase-1 levels likely reflects initiation (priming) of the inflammasome rather than its full activation. The inflammasome activation process occurs in two stages: the priming phase involves increased expression of its components (e.g., NLRP3, pro-caspase-1) dependent on NF-κB signaling, whereas full activation requires a second signal leading to caspase-1 cleavage and formation of an active inflammasome complex. On the other hand, voluntary wheel running in ovariectomized mice decreased *Il-1b* gene expression and induced the upregulation of NLRP3 protein, with no significant changes observed in the sham-operated mice. Other studies have shown that ovariectomy can lead to activation of the NLRP3 inflammasome, promoting increased production of proinflammatory cytokines IL-1*β* and IL-18, activation of caspase-1, upregulation of TLR2, TLR4, and NF-κB. This consequently leads to neuroinflammatory changes in the hippocampus and contributes to mood disturbances in ovariectomized females (Xu et al., 2016). Importantly, regular physical exercise has been demonstrated to alleviate these effects by suppressing NLRP3 inflammasome activation in the hippocampus (Wang et al., 2016). The discrepancy between elevated pro-IL-18 levels and reduced IL-1β expression may indicate partial activation of the NLRP3 inflammasome. Physical activity can induce a mild inflammatory response that increases inflammasome precursors, like pro-IL-18, without fully activating the inflammasome or triggering cytokine maturation (Gleeson et al., 2011). This response helps maintain immune surveillance while preventing excessive neuroinflammation. Our findings suggest that moderate physical activity promotes a balanced, anti-inflammatory state in hypothalamic inflammasome activity.

In summary, both sham-operated and ovariectomized mice exhibited similar expression patterns for most proteins and genes related to neuroinflammatory response following voluntary wheel running. The notable differences were in the *Il-1b* and *Casp1* gene expression levels between the sham and ovariectomized mice, but these changes were not reflected at the protein level.

We demonstrated that ovariectomy in middle-aged mice resulted in discrepancies in body mass and energy metabolism, whereas voluntary wheel running mitigated these effects. Furthermore, ovariectomy reduced the ERα-to-ERβ ratio, whereas voluntary wheel running increased hypothalamic ERβ levels. The voluntary wheel running induced changes in appetite-related peptides, which were not observed in females that underwent ovariectomy. Moreover, neuroinflammatory response within the hypothalamus following voluntary wheel running was comparable between sham-operated and ovariectomized females. Thus, it may be hypothesized that preserving ovarian function, even without changes in circulating E2 levels, may be necessary for exercise-induced changes in appetite-regulating factors. Our results further support the assumption that CNS exercise-induced effects on appetite and neuroinflammatory regulation seem to be modulated in an ERs level-dependent manner. These findings may contribute to the development of physical activity strategies to mitigate neuroinflammation and appetite dysregulation in postmenopausal women.

## Data Availability

The original contributions presented in the study are publicly available. This data can be found here: https://doi.org/10.18150/GELASZ.

## Supplementary material

SUPPLEMENTARY MATERIAL S1 Characteristics of standard chow, primary antibodies, and primers used in the experiment.

Supplementary material S2 Original full-length images of western blotting gels and, protein bands.

Supplementary material S3 Statistical analysis results.

## Glossary

Agrp: agouti-related protein
CCK: cholecystokinin
Cart: cocaine and amphetamine regulated transcript
CNS: central nervous system
Cckar: cholecystokinin receptor
E2: 17-β-estradiol
ER: estrogen receptor
FER: feeding efficiency ratio
Ghsr: ghrelin receptor
Glp1r: glucagon-like peptide-1 receptor
IL: interleukin
Lepr: leptin receptor
NF-κB: nuclear factor kappa-light-chain-enhancer of activated B cells
NLRP3: nucleotide-binding oligomerization domain-like receptor (NLR) family pyrin domain containing 3
Npy: neuropeptide Y
OVX: bilateral ovariectomy
Pomc: proopiomelanocortin
SHAM: sham operation
TLR: toll-like receptor
TNF: tumor necrosis factor
VWR: voluntary wheel running
