# Supplementary material for: Effects of voluntary wheel running on appetite-regulating peptides and neuroinflammation in the hypothalamus of ovariectomized middle-aged mice
Source: Front Mol Neurosci. 2025 Dec 8;18:1698306. doi: 10.3389/fnmol.2025.1698306 (PMC12719451; doi:10.3389/fnmol.2025.1698306)
Supplement: SUPPLEMENTARY MATERIAL S1 — Characteristics of standard chow, primary antibodies, and primers used in the experiment. [file Data_Sheet_1.docx]

# **Supplementary material 1**

**Effects of voluntary wheel running on appetite-regulating peptides and neuroinflammation in the hypothalamus of ovariectomized middle-aged mice**

Mateusz Grabowski^1*^, Konstancja Grabowska^1^, Magdalena Kostka^1^, Natalia Pondel^1^, Andrzej Małecki^1^, Jarosław J Barski^2^, and Marta Nowacka-Chmielewska^1^

^1^Laboratory of Molecular Biology, Institute of Physiotherapy and Health Sciences, Academy of Physical Education, Katowice, Poland

^2^Department of Physiology, Faculty of Medical Sciences in Katowice, Medical University of Silesia, Katowice, Poland

*** Corresponding author:** m.grabowski@awf.katowice.pl

**keywords:** appetite peptides, hypothalamus, neuroinflammation, inflammasome NLRP3, voluntary wheel running, physical activity, ovariectomy, menopause

**Table S1.1.** Characteristics of the standard chow (Labofeed B, Wytwórnia Pasz Morawki) used in the experiment.

| **Labofeed B composition (per kg)** | | | |
| --- | --- | --- | --- |
| Protein (g) | 175 | Total calories (kcal) | 2747 |
| *Soybean meal (%)* | 8 | Protein (kcal, %) | 25 |
| *Potato protein (%)* | 3 | Fat (kcal, %) | 8 |
| *Feed yeast (%)* | 3 | Carbohydrates (kcal, %) | 67 |
| Fat (g) | 2.8 |  |  |
| Fiber (g) | 70 |  |  |
| Starch (g) | 330 |  |  |
| Ash (g) | 57 |  |  |
| Calcium (g) | 9.5 |  |  |
| Phosphorus (g) | 6.5 |  |  |
| Magnesium (g) | 3 |  |  |
| Potassium (g) | 7.5 |  |  |
| Sodium (g) | 1.9 |  |  |
| Sulfur (g) | 1.9 |  |  |
| Iron (mg) | 144 |  |  |
| Manganese (mg) | 50 |  |  |
| Zink (mg) | 50 |  |  |
| Copper (mg) | 11 |  |  |
| Iodine (mg) | 0.2 |  |  |
| Selenium (mg) | 0.4 |  |  |
| Vitamin A (UI) | 12000 |  |  |
| Vitamin D3 (UI) | 800 |  |  |
| Vitamin E (mg) | 78 |  |  |
| Vitamin K3 (mg) | 2.4 |  |  |
| Vitamin B1 (mg) | 8 |  |  |
| Vitamin B2 (mg) | 7 |  |  |
| Vitamin B6 (mg) | 11 |  |  |
| Vitamin B12 (mg) | 42 |  |  |
| Pantothenic acid (mg) | 25 |  |  |
| Folic acid (mg) | 2 |  |  |
| Biotin (mg) | 0.3 |  |  |

**Table S1.2.** Producers, catalog numbers of primary antibodies, blocking buffers, antibodies diluents, dilutions of primary and secondary antibodies.

| **Primary antibody** | **Producer, catalog number** | **Dilution of primary antibody** | **Blocking buffer** | **Primary antibody diluent** | **Secondary antibody diluent*** | **Dilution of secondary antibody*** |
| --- | --- | --- | --- | --- | --- | --- |
| **anti-Cckar** | Bioss, bs-11514R | 1:1000 | 1x TBST with 1% BSA | 1x TBST with 1% BSA | 1x TBST with 1% BSA | 1:5000 |
| **anti-ERα** | HUABIO, ER1803-83 | 1:1000 | 1x TBST with 5% NFDM | 1x TBST with 5% NFDM | 1x TBST with 5% NFDM | 1:4000 |
| **anti-ERβ** | Invitrogen, PA1-310B | 1:1000 | 1x TBST with 5% NFDM | 1x TBST with 5% NFDM | 1x TBST with 5% NFDM | 1:4000 |
| **anti-Ghsr** | Invitrogen, PA5-28752 | 1:1000 | 1x TBST with 5% NFDM | 1x TBST with 5% NFDM | 1x TBST with 5% NFDM | 1:4000 |
| **anti-Glp-1r** | Bioss, bs-1559R | 1:1000 | 1x casein/ H_2_O | 1x casein/ H_2_O | 1x casein/ H_2_O | 1:5000 |
| **anti-Lepr** | Invitrogen, PA5-119252 | 1:750 | 1x casein/ H_2_O | 1x casein/ H_2_O | 1x casein/ H_2_O | 1:4000 |
| **anti-NF-κb  p65** | Abcam, ab16502 | 1:1000 | 1x TBST with 5% BSA | 1x TBST with 5% BSA | 1x TBST with 5% BSA | 1:4000 |
| **anti-TLR4** | Invitrogen, PA5-23124 | 1:1000 | 1x casein/ H_2_O | 1x casein/ H_2_O | 1x casein/ H_2_O | 1:5000 |
| **anti-NLRP3** | Abcam, ab270449 | 1:750 | 1x TBST with 5% NFDM | 1x TBST with 5% NFDM | 1x TBST with 5% NFDM | 1:4000 |
| **anti-Pomc** | Abcam, ab254257 | 1:1000 | 1x TBST with 5% NFDM | 1x TBST with 5% NFDM | 1x TBST with 5% NFDM | 1:4000 |
| **anti-pro Caspase-1** | Abcam, ab179515 | 1:1000 | 1x TBST with 5% NFDM | 1x TBST with 5% NFDM | 1x TBST with 5% NFDM | 1:4000 |
| **anti-pro-IL-1β** | Abcam, 254360 | 1:1000 | 1x TBST with 5% NFDM | 1x TBST with 5% NFDM | 1x TBST with 5% NFDM | 1:4000 |
| **anti-pro-IL-18** | Cell Signaling, 57058S | 1:1000 | 1x casein/ H_2_O | 1x casein/ H_2_O | 1x casein/ H_2_O | 1:5000 |

*The secondary antibody was used: (11-035-003, Jackson Immunoresearch Laboratories). BSA – bovine serum albumin, TBST – tris buffered saline with Tween 20, NFDM – non-fat dry milk

**Table S1.3.** List of primers used in the experiment.

| **Gene** | **Sequence 5’ 🡪 3’** |
| --- | --- |
| ***Gapdh*** | F:CAACTCCCTCAAGATTGTCAGCAA R:GGCATGGACTGTGGTCATGA |
| ***Agrp*** | F: ACCTTAGGGAGGCACCTCAT R: AGCAACATTGCAGTCAGCAT |
| ***Cart*** | F: ACGAGAAGGAGCTGCCAAG, ﻿  R: GCTCTCCAGCGTCACACAT |
| ***Pomc*** | F: GAAGATGCCGAGATTCTGCT  R: CTCCAGCGAGAGGTCGAGTT |
| ***Lepr*** | F: CCTGGGCACAAGGACTGAAT R: TGGACTGTTGGGAAGTTGGTA |
| ***Npy*** | F: CCTGGGCACAAGGACTGAAT  R: TGGACTGTTGGGAAGTTGGTA |
| ***Rela*** | F: GCCTCTGGCGAATGGCTTTA  R: TGCTTCGGCTGTTCGATGAT |
| ***Relb*** | F: CTTGGGTTCCAGTGACCTCTC  R: TGGTCCTGGAGACCGTTAGT |
| ***Nlrp3*** | F: GCAGAGCCTACAGTTGGGTG  R: ACGCCTACCAGGAAATCTCG |
| ***Casp1*** | F: GGCACATTTCCAGGACTGACTG  R: GCAAGACGTGTACGAGTGGTTG |
| ***Il-1b*** | F: GTTCATCTCGGAGCCTGTAGTG  R: TGGACCTTCCAGGATGAGGACA |
| ***Il-18*** | F: GACAGCCTGTGTTCGAGGATATG  R: TGTTCTTACAGGAGAGGGTAGAC |
| ***Il-6*** | F: CCACTTCACAAGTCGGAGGC  R: GAATTGCCATTGCACAACTCTT |
| ***Tnfa*** | F: CCACCACGCTCTTCTGTCTAC  R: GCCATTTGGGAACTTCTCATCCC |

F – forward, R - reverse
