# Supplementary material for: Effects of voluntary wheel running on appetite-regulating peptides and neuroinflammation in the hypothalamus of ovariectomized middle-aged mice
Source: Front Mol Neurosci. 2025 Dec 8;18:1698306. doi: 10.3389/fnmol.2025.1698306 (PMC12719451; doi:10.3389/fnmol.2025.1698306)
Supplement: Supplementary material S2 — Original full-length images of western blotting gels and, protein bands. [file Data_Sheet_2.docx]

# **Supplementary materials 2**

**Effects of voluntary wheel running on appetite-regulating peptides and neuroinflammation in the hypothalamus of ovariectomized middle-aged mice.**

Mateusz Grabowski^1*^, Konstancja Grabowska^1^, Magdalena Kostka^1^, Natalia Pondel^1^, Andrzej Małecki^1^, Jarosław J Barski^2^, and Marta Nowacka-Chmielewska^1^

^1^Laboratory of Molecular Biology, Institute of Physiotherapy and Health Sciences, Academy of Physical Education, Katowice, Poland

^2^Department of Physiology, Faculty of Medical Sciences in Katowice, Medical University of Silesia, Katowice, Poland

*** Corresponding author:** m.grabowski@awf.katowice.pl

**keywords:** appetite peptides, hypothalamus, neuroinflammation, inflammasome NLRP3, voluntary wheel running, physical activity, ovariectomy, menopause

**Table S2.1.** The percentage distribution of the estrus cycle stages of middle-aged animals on the operation day (Day 0) and seven weeks after operations (Day 49).

|  | **Day 0** | **Day 49** | |
| --- | --- | --- | --- |
|  | **ALL MICE** | **SHAM** | **OVX** |
| **PROESTRUS** | 17.5% | 10% | - |
| **ESTRUS** | 25% | 20% | - |
| **METESTRUS/DIESTRUS** | 57.5% | 70% | 100% |

SHAM - sham operation, OVX - bilateral ovariectomy. n = 40/all mice, n = 20 per SHAM/OVX.

**Table S2.2.** Results of the Friedman test followed by Dunn's multiple comparisons test of the average weekly distance covered in the active phase during six weeks of voluntary wheel running.

|  | **SHAM-VWR**  **(p-value)** | **OVX-VWR**  **(p-value)** |
| --- | --- | --- |
| **Friedman test** | < 0.0001 | < 0.0001 |
| **Dunn's multiple comparisons test** |  |  |
| **1 vs. 2 week** | > 0.9999 | < 0.0001 |
| **1 vs. 3 week** | > 0.9999 | < 0.0001 |
| **1 vs. 4 week** | 0.4398 | < 0.0001 |
| **1 vs. 5 week** | < 0.0001 | < 0.0001 |
| **1 vs. 6 week** | < 0.0001 | < 0.0001 |

n = 6 - 7 per group. SHAM - sham operation, OVX - bilateral ovariectomy, VWR - voluntary wheel running.

**Table S2.3.** The average body mass (g) over the seven-week experimental period. Results of ordinary one-way ANOVA of the average body mass in each week.

|  | **0 week** | **1 week** | **2 week** | **3 week** | **4 week** | **5 week** | **6 week** | **7 week** |
| --- | --- | --- | --- | --- | --- | --- | --- | --- |
| **SHAM-SED** | 25.73 ± 1.91 | 26.47 ± 1.69 | 26.74 ± 1.29 | 27.03 ± 1.66 | 26.62 ± 1.69 | 26.92 ± 1.89 | 27.05 ± 1.53 | 27.51 ± 2.02 |
| **OVX-SED** | 25.69 ± 2.33 | 25.29 ± 2.12 | 27.50 ± 1.99 | 28.26 ± 2.36 | 27.85 ± 2.37 | 28.29 ± 2.66 | 29.01 ± 2.94 | 30.22 ± 3.15 |
| **SHAM-VWR** | 26.38 ± 2.94 | 25.98 ± 1.73 | 25.75 ± 1.47 | 26.11 ± 1.17 | 26.01 ± 1.78 | 26.29 ± 2.34 | 26.79 ± 1.72 | 27.26 ± 2.13 |
| **OVX-VWR** | 24.77 ± 2.19 | 25.62 ± 1.73 | 27.04 ± 0.97 | 27.51 ± 1.27 | 26.59 ± 0.93 | 26.56 ± 1.21 | 26.85 ± 0.95 | 27.50 ± 1.3 |
| **p - value** | 0.5132 | 0.5251 | 0.0726 | 0.0503 | 0.1449 | 0.1639 | 0.0418 | 0.0168 |

Values ​​are presented as mean ± SD (g). SHAM - sham operation, OVX - bilateral ovariectomy, SED – sedentary, VWR – voluntary wheel running. n = 10 per group.

**Table S2.4.** Results of Repeated measures (RM) one-way ANOVA followed by Tukey's multiple comparisons test of the average body mass over the seven-week experimental period.

|  | **SHAM-SED**  **(p-value)** | **OVX-SED**  **(p-value)** | **SHAM-VWR**  **(p-value)** | **OVX-VWR**  **(p-value)** |
| --- | --- | --- | --- | --- |
| **RM One-way ANOVA** | < 0.0001 | < 0.0001 | < 0.0001 | 0.0008 |
| **Tukey's multiple comparisons** |  | | | |
| **0 vs. 1 week** | 0.8236 | 0.9771 | 0.9943 | 0.7172 |
| **0 vs. 2 week** | 0.5170 | 0.0120 | 0.9626 | 0.0302 |
| **0 vs. 3 week** | 0.4047 | 0.0480 | 0.9998 | 0.0026 |
| **0 vs. 4 week** | 0.7669 | 0.0004 | 0.9981 | 0.1220 |
| **0 vs. 5 week** | 0.5148 | 0.0016 | >0.9999 | 0.1649 |
| **0 vs. 6 week** | 0.4253 | 0.0008 | 0.9956 | 0.1727 |
| **0 vs. 7 week** | 0.0607 | 0.0001 | 0.9409 | 0.0827 |

n = 10 per group. SHAM - sham operation, OVX - bilateral ovariectomy, SED – sedentary, VWR - voluntary wheel running.

**Table S2.5.** Results of the two-way ANOVA analysis of middle-aged mice's hypothalamus protein expression level seven weeks post-operation.

|  | **Intervention (VWR)** | | **Operation** | | **Interaction (intervention x operation)** | |
| --- | --- | --- | --- | --- | --- | --- |
|  | F (DFn, DFd) | p-value | F (DFn, DFd) | p-value | F (DFn, DFd) | p-value |
| **ERα** | F (1, 16) = 0.96 | 0.3430 | F (1, 16) = 1.56 | 0.2299 | F (1, 16) = 0.01 | 0.9138 |
| **ERβ** | F (1, 16) = 6.27 | 0.0235 | F (1, 16) = 3.25 | 0.0902 | F (1, 16) = 2.86 | 0.1105 |
| **ERα/ERβ ratio** | F (1, 16) = 0.72 | 0.4094 | F (1, 16) = 5.34 | 0.0345 | F (1, 16) = 1.25 | 0.2809 |
| **Cckar** | F (1, 16) = 5.29 | 0.0352 | F (1, 16) = 0.04 | 0.8367 | F (1, 16) = 3.36 | 0.0857 |
| **Glp1r** | F (1, 16) = 5.13 | 0.0378 | F (1, 16) = 0.56 | 0.4645 | F (1, 16) = 2.08 | 0.1684 |
| **Ghsr** | F (1, 16) = 0.33 | 0.5730 | F (1, 16) = 1.72 | 0.2083 | F (1, 16) = 9.53 | 0.0071 |
| **Pomc** | F (1, 16) = 0.12 | 0.7378 | F (1, 16) = 0.76 | 0.3970 | F (1, 16) = 0.12 | 0.7321 |
| **Lepr** | F (1, 16) = 4.28 | 0.0551 | F (1, 16) = 0.21 | 0.6514 | F (1, 16) = 9.88 | 0.0063 |
| **NLRP3** | F (1, 16) = 5.89 | 0.0274 | F (1, 16) = 1.33 | 0.2658 | F (1, 16) = 0.06 | 0.8172 |
| **Pro-caspase 1** | F (1, 16) = 19.06 | 0.0005 | F (1, 16) = 1 | 0.7563 | F (1, 16) = 2.32 | 0.1472 |
| **Pro-IL-1β** | F (1, 16) = 1.8 | 0.1990 | F (1, 16) = 0.41 | 0.5326 | F (1, 16) = 0.05 | 0.8206 |
| **Pro-IL-18** | F (1, 16) = 18.71 | 0.0005 | F (1, 16) = 0.03 | 0.8597 | F (1, 16) = 15.44 | 0.0012 |
| **TLR4** | F (1, 16) = 20.74 | 0.0003 | F (1, 16) = 1.52 | 0.2353 | F (1, 16) = 0.25 | 0.6256 |
| **NF-κB p65** | F (1, 16) = 0.03 | 0.8615 | F (1, 16) = 1.09 | 0.3115 | F (1, 16) = 0.09 | 0.7704 |

n = 5 per group. DFn - numerator degrees of freedom, DFd - the denominator degrees of freedom, VWR - voluntary wheel running.

**Table S2.6.** Results of the two-way ANOVA analysis of the middle-aged mice's hypothalamus gene expression level seven weeks post-operation.

|  | **Intervention (VWR)** | | **Operation** | | **Interaction (intervention x operation)** | |
| --- | --- | --- | --- | --- | --- | --- |
|  | F (DFn, DFd) | p-value | F (DFn, DFd) | p-value | F (DFn, DFd) | p-value |
| ***Cart*** | F (1, 19) = 0.32 | 0.5760 | F (1, 19) = 1.66 | 0.2131 | F (1, 19) = 0.02 | 0.9025 |
| ***Pomc*** | F (1, 19) = 0.35 | 0.5606 | F (1, 19) = 4.47 | 0.0479 | F (1, 19) = 2.51 | 0.1300 |
| ***Agrp*** | F (1, 19) = 0.03 | 0.8543 | F (1, 19) = 0.01 | 0.9981 | F (1, 19) = 0.34 | 0.5667 |
| ***Npy*** | F (1, 20) = 0.59 | 0.4524 | F (1, 20) = 0.01 | 0.9691 | F (1, 20) = 0.99 | 0.3314 |
| ***Lepr*** | F (1, 19) = 0.15 | 0.6993 | F (1, 19) = 1.65 | 0.2146 | F (1, 19) = 5.15 | 0.0351 |
| ***Nlrp3*** | F (1, 19) = 0.01 | 0.9524 | F (1, 19) = 0.36 | 0.5549 | F (1, 19) = 0.85 | 0.3686 |
| ***Casp1*** | F (1, 19) = 4.12 | 0.0567 | F (1, 19) = 4.56 | 0.0459 | F (1, 19) = 5.62 | 0.0285 |
| ***Il-1b*** | F (1, 18) = 15.76 | 0.0009 | F (1, 18) = 0.59 | 0.4528 | F (1, 18) = 0.02 | 0.8957 |
| ***Il-18*** | F (1, 19) = 5.07 | 0.0363 | F (1, 19) = 1.1 | 0.3073 | F (1, 19) = 0.07 | 0.7875 |
| ***Rela*** | F (1, 20) = 0.01 | 0.9426 | F (1, 20) = 1.1 | 0.3079 | F (1, 20) = 0.1 | 0.7566 |
| ***Relb*** | F (1, 19) = 0.02 | 0.9007 | F (1, 19) = 0.85 | 0.3671 | F (1, 19) = 0.41 | 0.5313 |
| ***Il-6*** | F (1, 20) = 0.02 | 0.8989 | F (1, 20) = 0.05 | 0.8197 | F (1, 20) = 0.61 | 0.4456 |
| ***Tnf*** | F (1, 12) = 1.1 | 0.3148 | F (1, 12) = 0.5 | 0.4938 | F (1, 12) = 1.93 | 0.1899 |

n = 5 - 7 per group. DFn - numerator degrees of freedom, DFd - the denominator degrees of freedom, VWR - voluntary wheel running.

**Table S2.7.** Results of the two-way ANOVA analysis of middle-aged mice's serum leptin, ghrelin, and cholecystokinin (CCK) seven weeks post-operation.

|  | **Intervention (VWR)** | | **Operation** | | **Interaction (intervention x operation)** | |
| --- | --- | --- | --- | --- | --- | --- |
|  | F (DFn, DFd) | p-value | F (DFn, DFd) | p-value | F (DFn, DFd) | p-value |
| **Leptin** | F (1, 28) = 6.82 | 0.0143 | F (1, 28) = 0.08 | 0.7832 | F (1, 28) = 0.01 | 0.9113 |
| **Ghrelin** | F (1, 21) = 0.16 | 0.6913 | F (1, 21) = 0.09 | 0.7708 | F (1, 21) = 0.07 | 0.7997 |
| **CCK** | F (1, 21) = 0.07 | 0.7970 | F (1, 21) = 1.39 | 0.2525 | F (1, 21) = 0.18 | 0.6733 |

n = 6 - 8 per group. DFn - numerator degrees of freedom, DFd - the denominator degrees of freedom, VWR - voluntary wheel running.
