# Supplementary material for: Effects of voluntary wheel running on appetite-regulating peptides and neuroinflammation in the hypothalamus of ovariectomized middle-aged mice
Source: Front Mol Neurosci. 2025 Dec 8;18:1698306. doi: 10.3389/fnmol.2025.1698306 (PMC12719451; doi:10.3389/fnmol.2025.1698306)
Supplement: Supplementary material S3 — Statistical analysis results. [file Data_Sheet_3.docx]

# Supplementary material 3

**Effects of voluntary wheel running on appetite-regulating peptides and neuroinflammation in the hypothalamus of ovariectomized middle-aged mice**

Mateusz Grabowski^1*^, Konstancja Grabowska^1^, Magdalena Kostka^1^, Natalia Pondel^1^, Andrzej Małecki^1^, Jarosław J Barski^2^, and Marta Nowacka-Chmielewska^1^

^1^Laboratory of Molecular Biology, Institute of Physiotherapy and Health Sciences, Academy of Physical Education, Katowice, Poland

^2^Department of Physiology, Faculty of Medical Sciences in Katowice, Medical University of Silesia, Katowice, Poland

*** Corresponding author:** m.grabowski@awf.katowice.pl

**keywords:** appetite peptides, hypothalamus, neuroinflammation, inflammasome NLRP3, voluntary wheel running, physical activity, ovariectomy, menopause


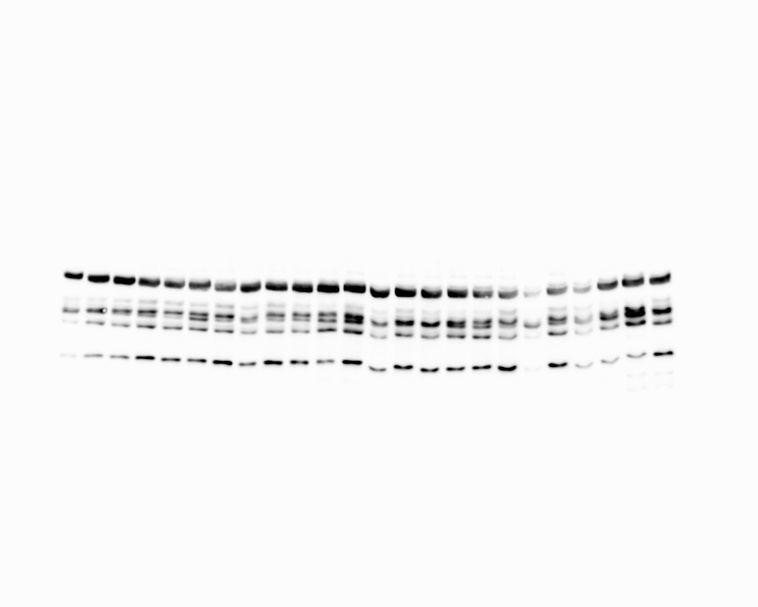


**Fig. S3.1** Chemiluminescence visualization of ERα bands on western blot membrane.


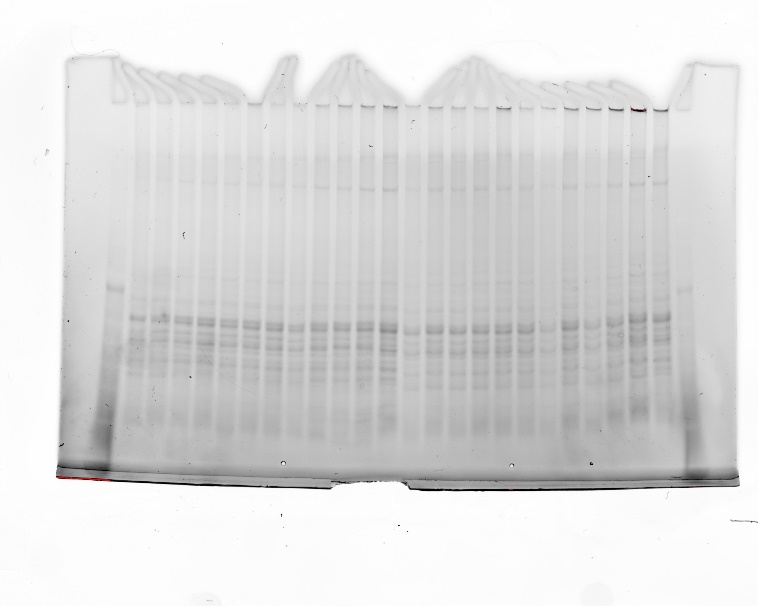


**Fig. S3.2** Visualization of total protein on polyacrylamide pre-cast gel (Bio-Rad) after electrophoresis under UV light as loading control to ERα.


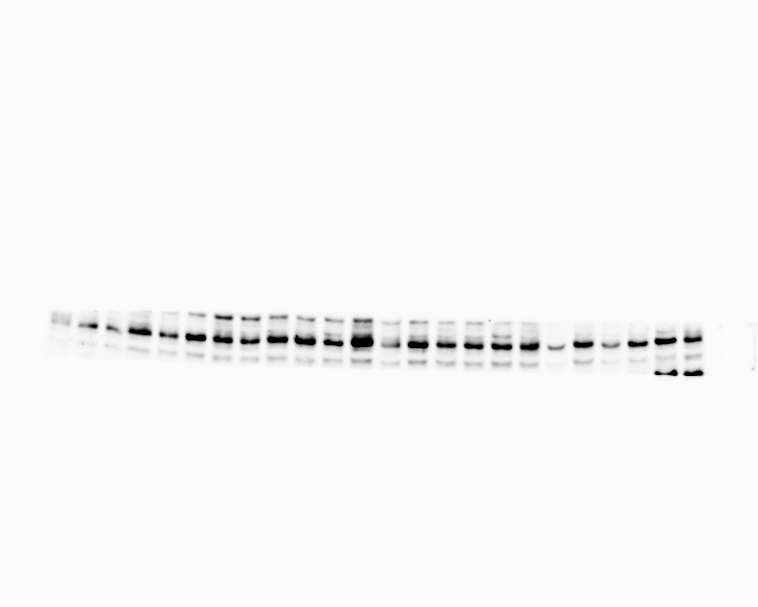


**Fig. S3.3** Chemiluminescence visualization of ERβ bands on western blot membrane.


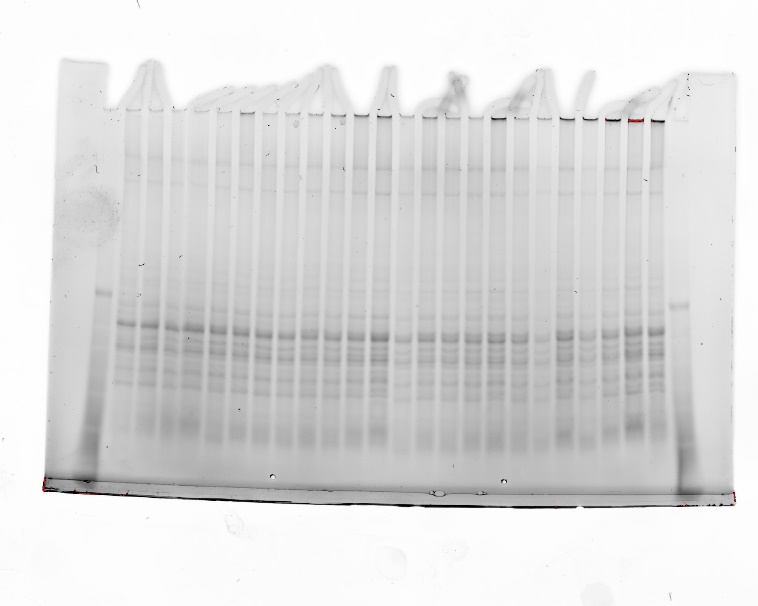


**Fig. S3.4** Visualization of total protein on polyacrylamide pre-cast gel (Bio-Rad) after electrophoresis under UV light as loading control to ERβ.


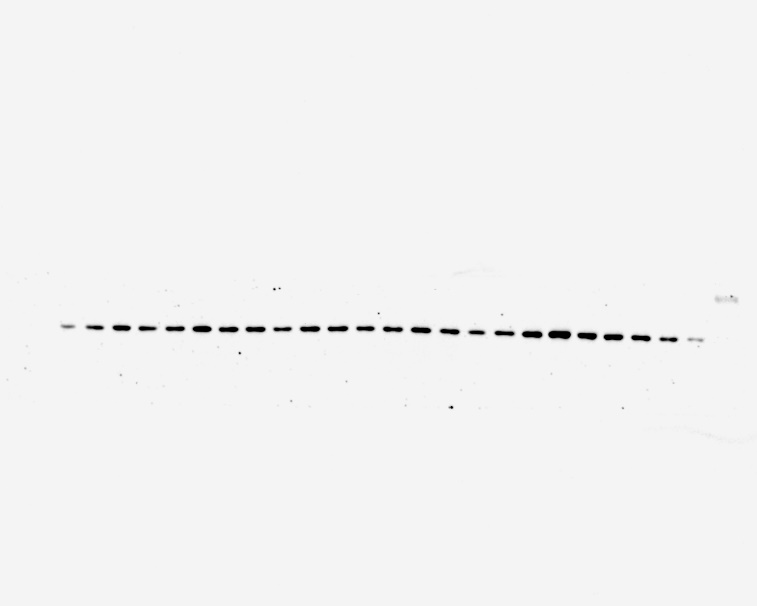


**Fig. S3.5** Chemiluminescence visualization of Glp1r bands on western blot membrane.


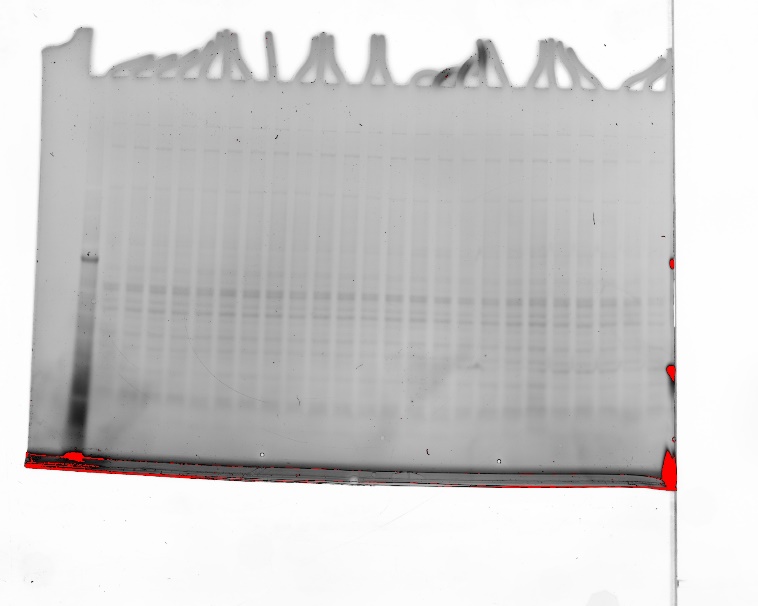


**Fig. S3.6** Visualization of total protein on polyacrylamide pre-cast gel (Bio-Rad) after electrophoresis under UV light as loading control to Glp1r.


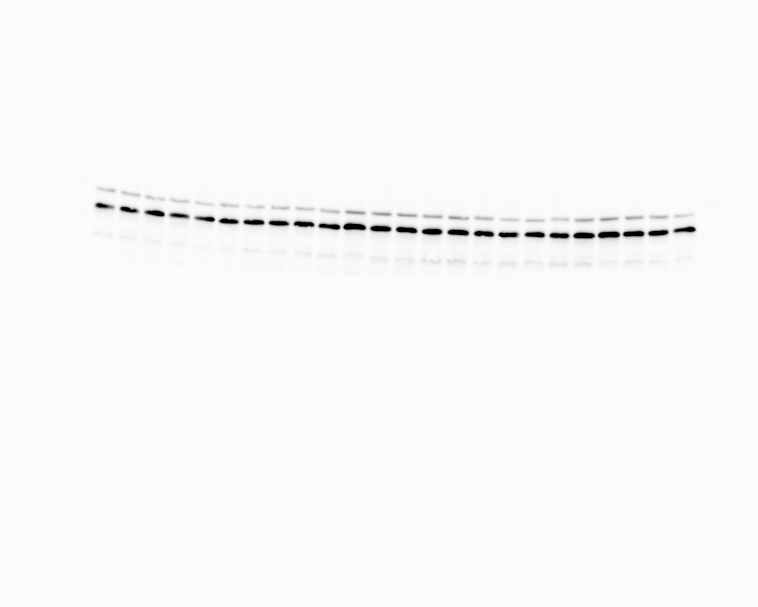


**Fig. S3.7** Chemiluminescence visualization of Cckar bands on western blot membrane.


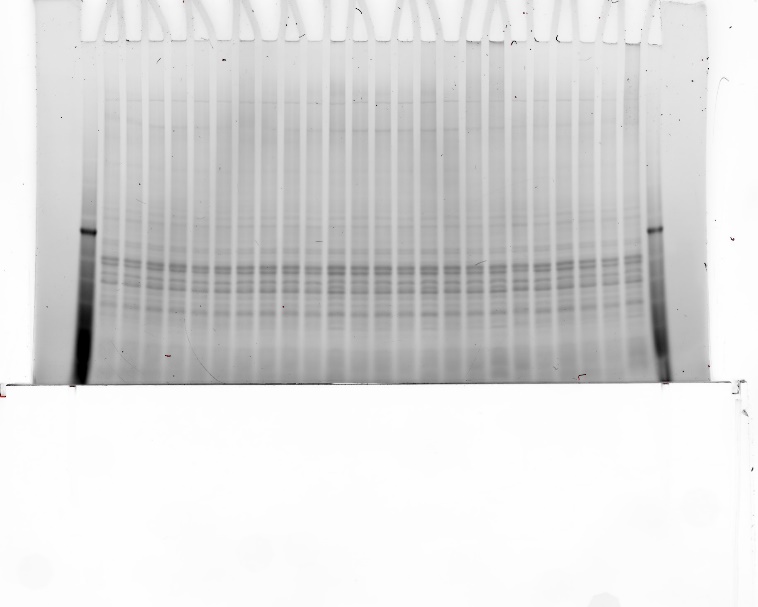


**Fig. S3.8** Visualization of total protein on polyacrylamide pre-cast gel (Bio-Rad) after electrophoresis under UV light as loading control to Cckar.


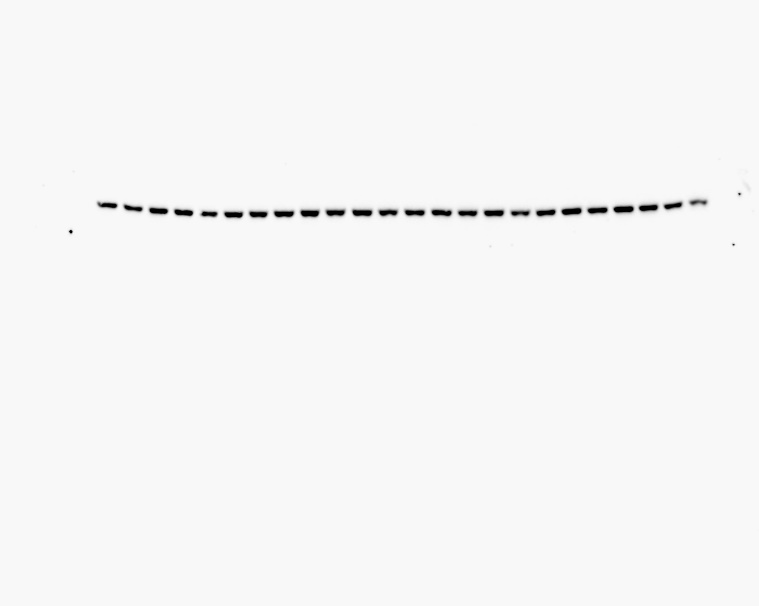


**Fig. S3.9** Chemiluminescence visualization of Ghsr bands on western blot membrane.


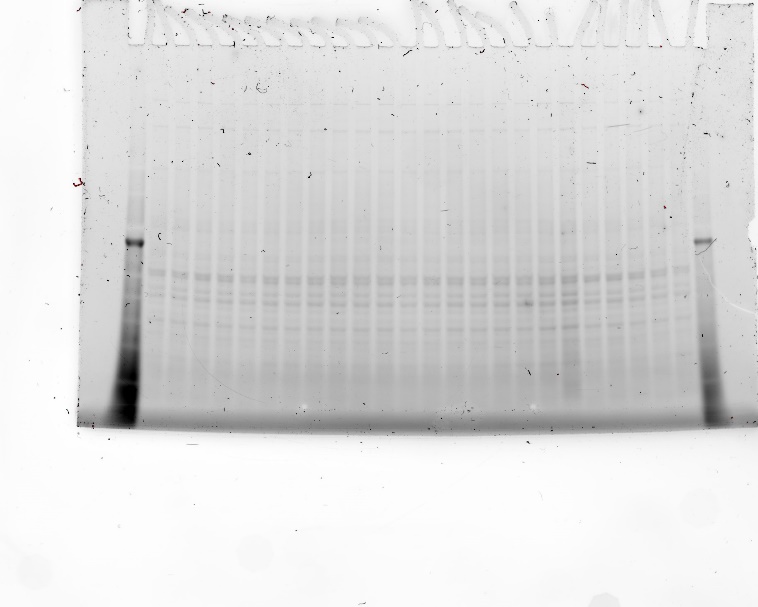


**Fig. S3.10** Visualization of total protein on polyacrylamide pre-cast gel (Bio-Rad) after electrophoresis under UV light as loading control to Ghsr.


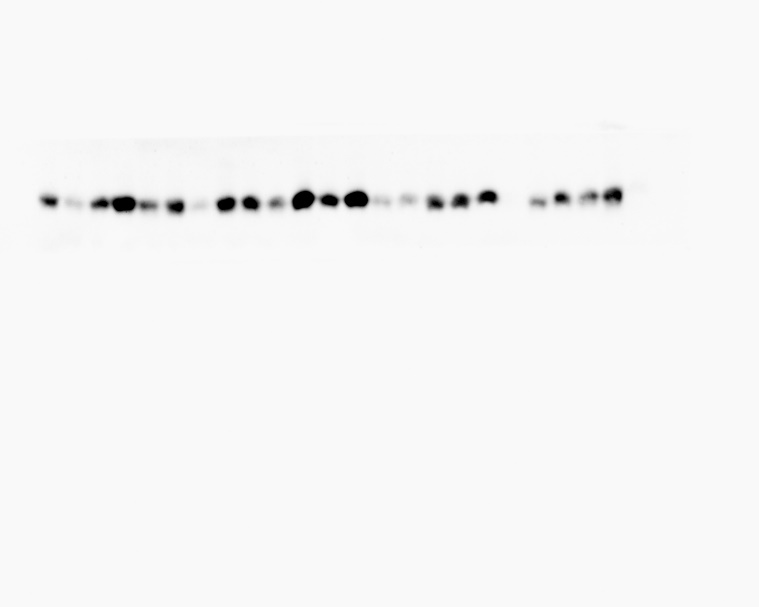


**Fig. S3.11** Chemiluminescence visualization of Pomc bands on western blot membrane.


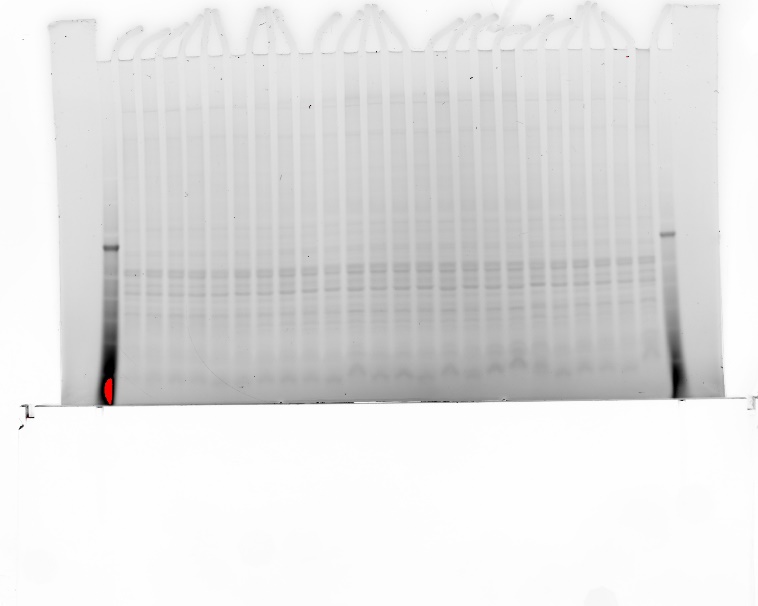


**Fig. S3.12** Visualization of total protein on polyacrylamide pre-cast gel (Bio-Rad) after electrophoresis under UV light as loading control to Pomc.


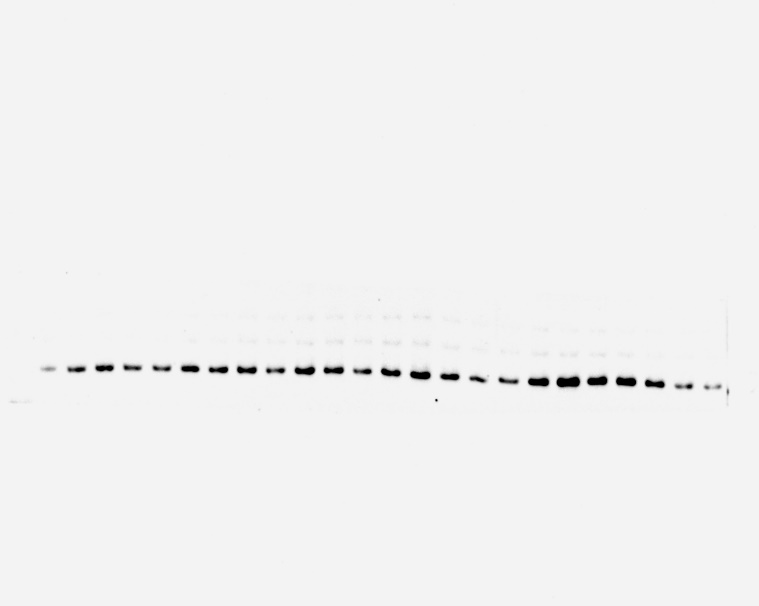


**Fig. S3.13** Chemiluminescence visualization of Lepr bands on western blot membrane.


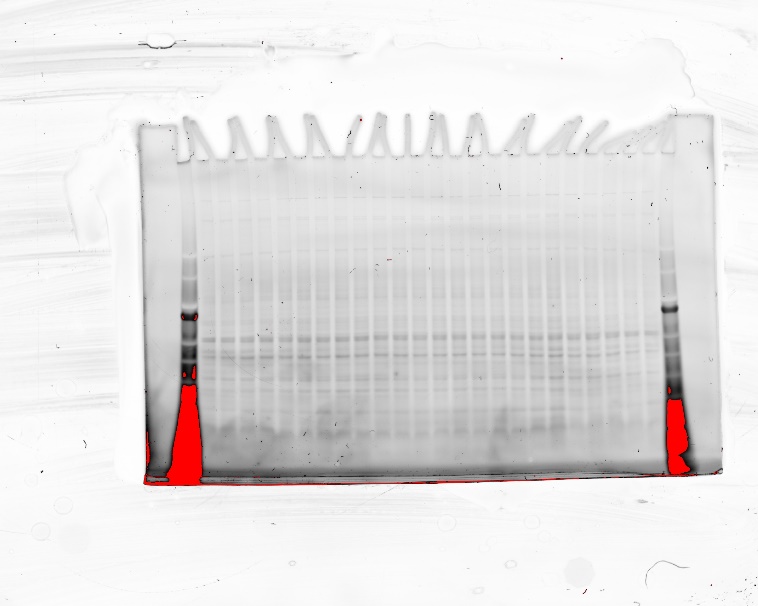


**Fig. S3.14** Visualization of total protein on polyacrylamide pre-cast gel (Bio-Rad) after electrophoresis under UV light as loading control to Lepr.


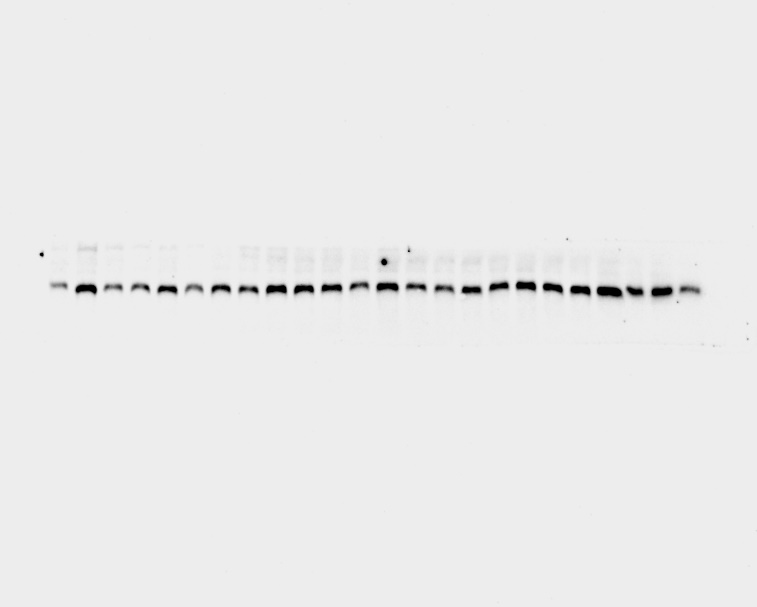


**Fig. S3.15** Chemiluminescence visualization of NLRP3 bands on western blot membrane.


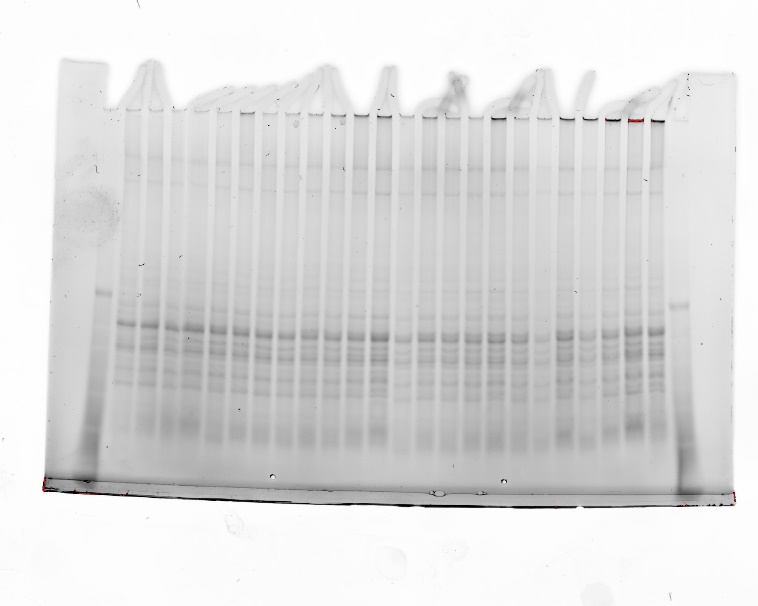


**Fig. S3.16** Visualization of total protein on polyacrylamide pre-cast gel (Bio-Rad) after electrophoresis under UV light as loading control to NLRP3.


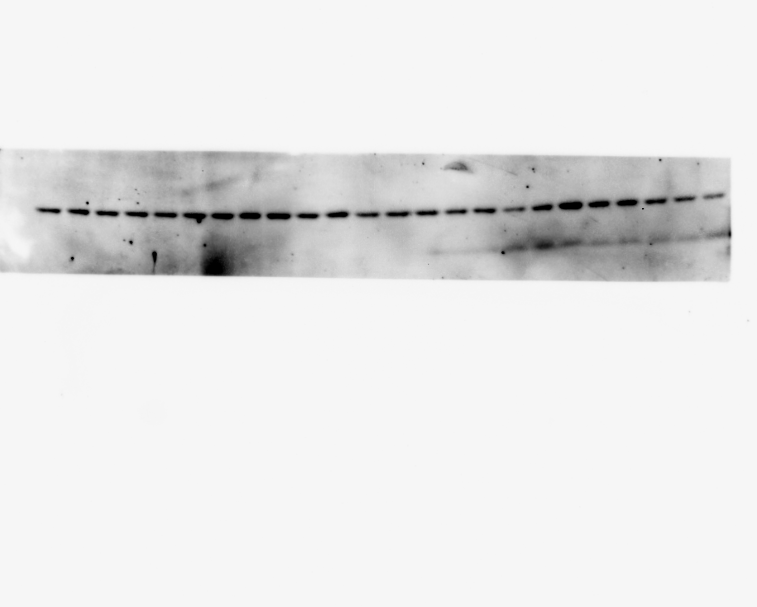


**Fig. S3.17** Chemiluminescence visualization of pro-IL-1β bands on western blot membrane.

##
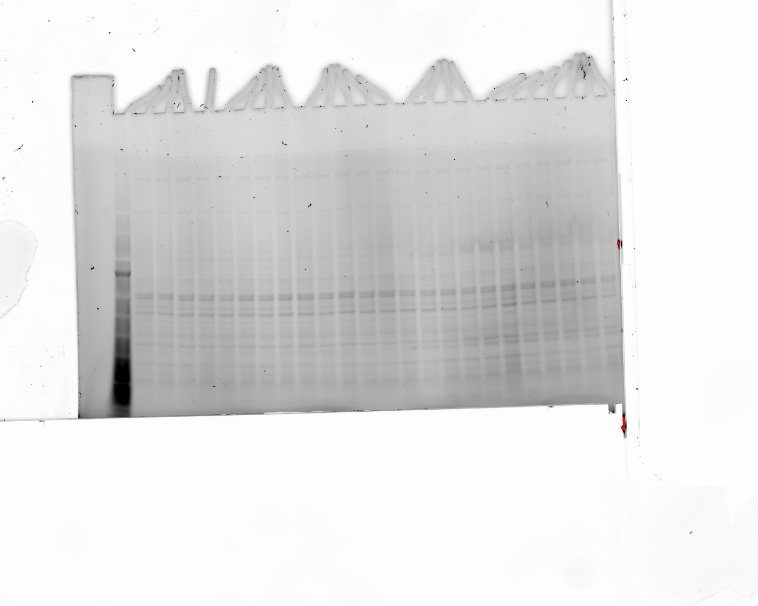


**Fig. S3.18** Visualization of total protein on polyacrylamide pre-cast gel (Bio-Rad) after electrophoresis under UV light as loading control to pro-IL-1β.


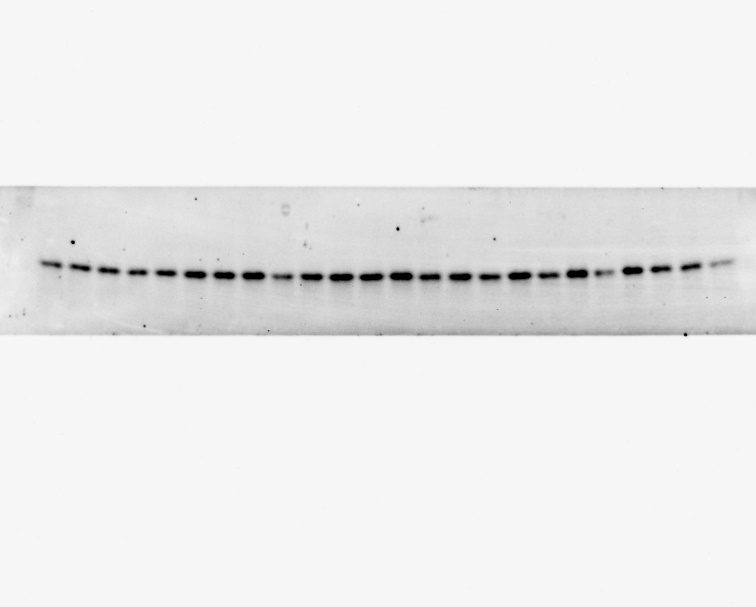


**Fig. S3.19** Chemiluminescence visualization of pro-IL-18 bands on western blot membrane.


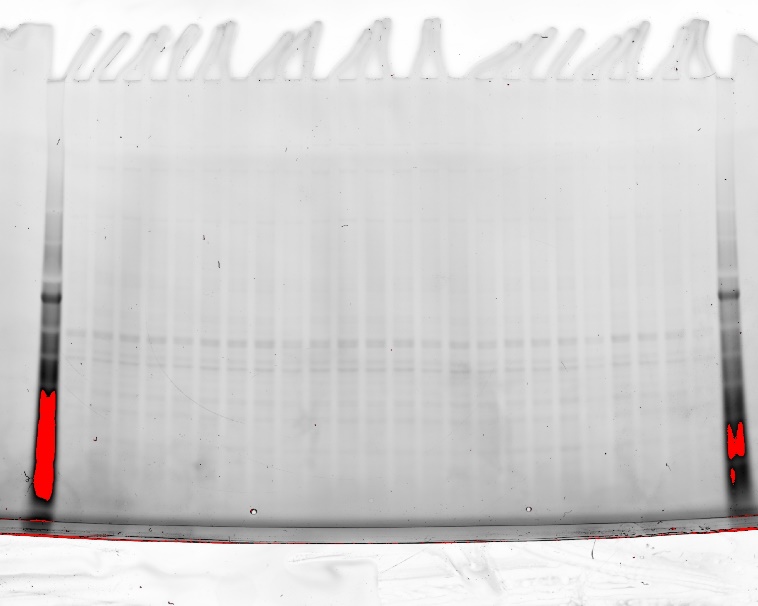


**Fig. S3.20** Visualization of total protein on polyacrylamide pre-cast gel (Bio-Rad) after electrophoresis under UV light as loading control to pro-IL-18.


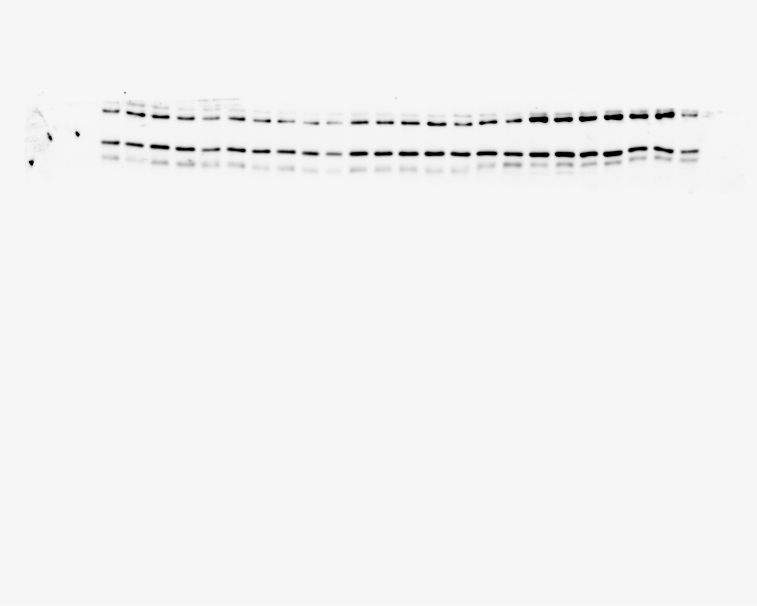


**Fig. S3.21** Chemiluminescence visualization of pro-caspase 1 bands on western blot membrane.


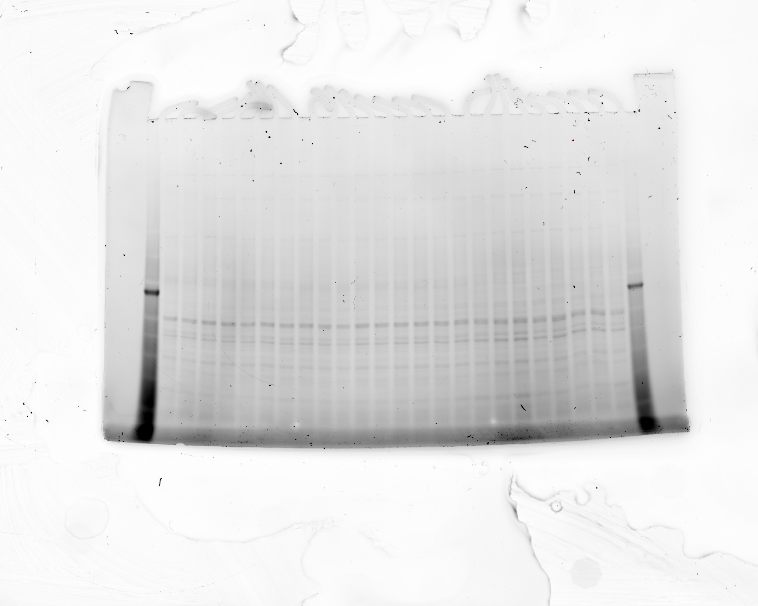


**Fig. S3.22** Visualization of total protein on polyacrylamide pre-cast gel (Bio-Rad) after electrophoresis under UV light as loading control to pro-caspase 1.


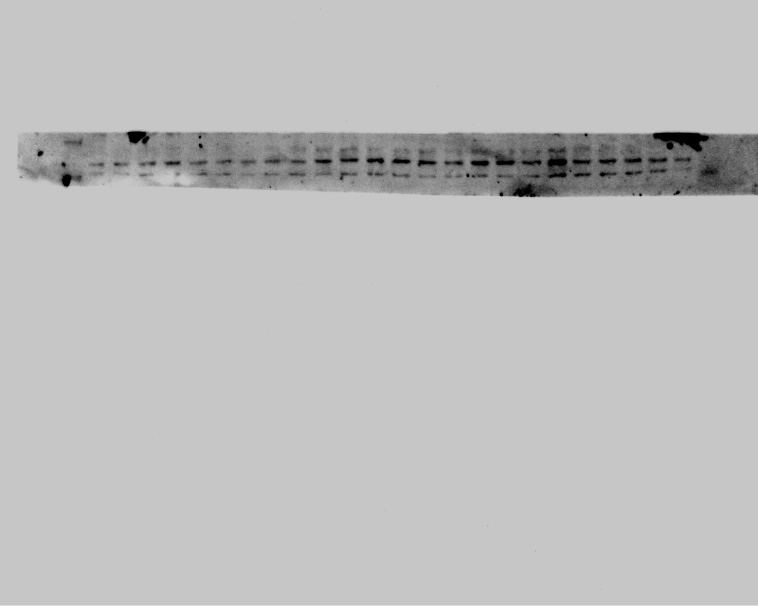


**Fig. S3.23** Chemiluminescence visualization of TLR4 bands on western blot membrane.


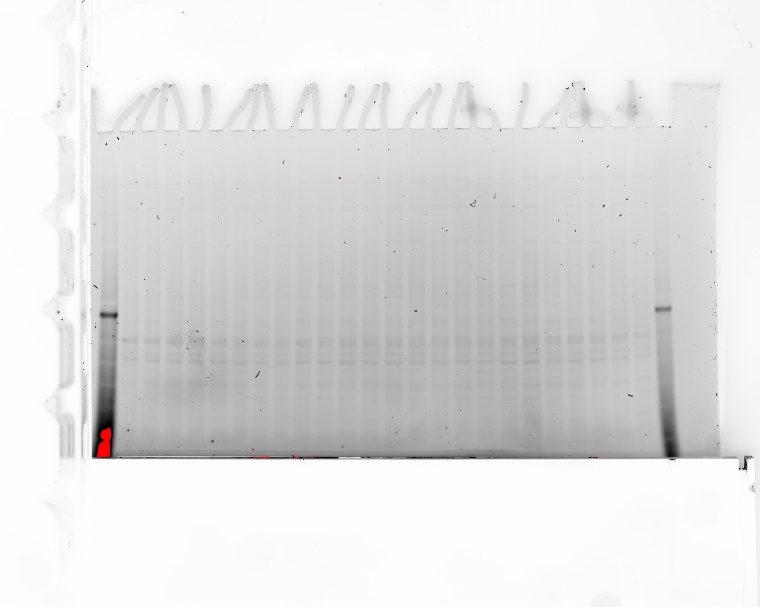


**Fig. S3.24** Visualization of total protein on polyacrylamide pre-cast gel (Bio-Rad) after electrophoresis under UV light as loading control to TLR4.


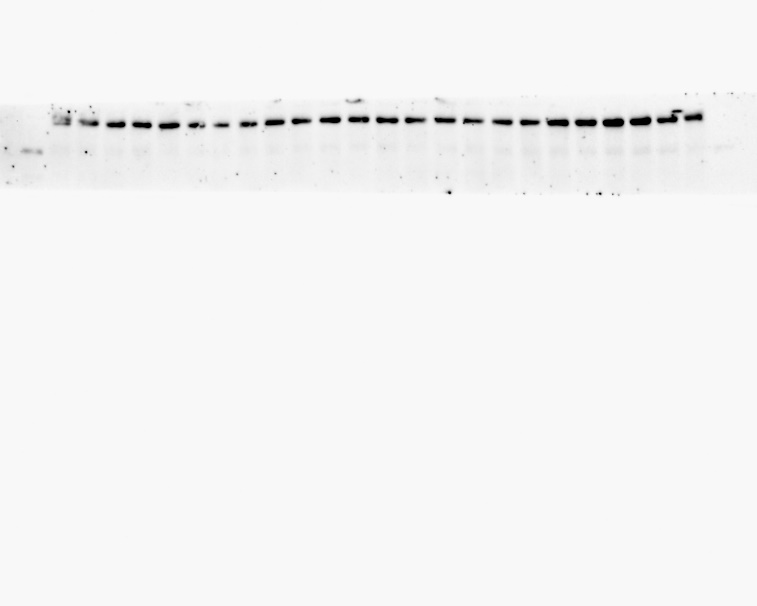


**Fig. S3.25** Chemiluminescence visualization of NF-κB p65 bands on western blot membrane.


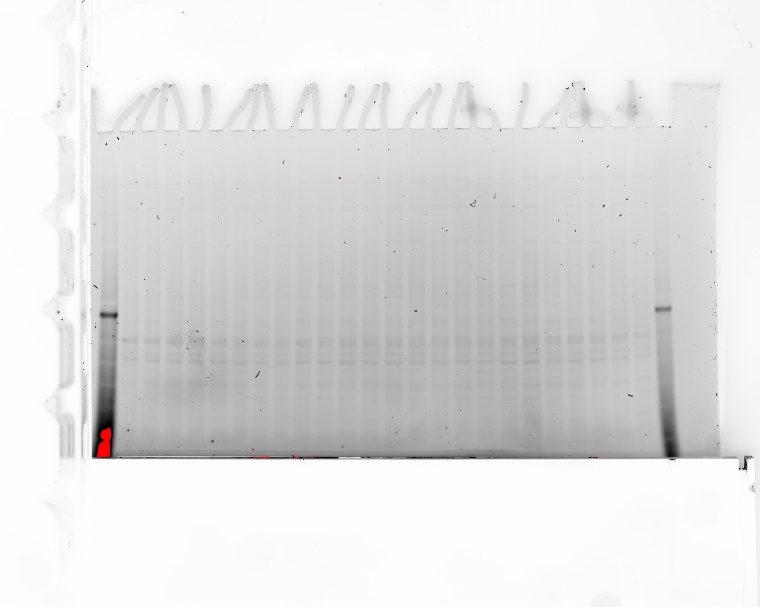


**Fig. S3.26** Visualization of total protein on polyacrylamide pre-cast gel (Bio-Rad) after electrophoresis under UV light as loading control to NF-κB p65.
